# Novel [^18^F]-labeled thiol for the labeling of Dha- or maleimide-containing biomolecules

**DOI:** 10.1186/s41181-022-00160-5

**Published:** 2022-04-06

**Authors:** Mylène Richard, Françoise Hinnen, Bertrand Kuhnast

**Affiliations:** Université Paris-Saclay, CEA, CNRS, Inserm, BioMaps, 91401 Orsay, France

**Keywords:** PET, Fluorine-18, Prosthetic group, Thiols, Dha, Maleimide

## Abstract

**Background:**

Prosthetic approach for the radiolabeling of biologics with fluorine-18 is a robust strategy and has been employed for many years. It requires fast, biocompatible and selective reactions suited to these fragile molecules. Michael addition of a nucleophilic thiol moiety on α,β-unsaturated carbonyl entities is an interesting compromise between simplicity of preparation of the prosthetic reagent and control of the selectivity of the addition. The α,β-unsaturated carbonyl entity of the biologic can easily be generated by addition of a maleimide function using adequate heterobifunctional linkers or generated by selective modification of a cysteine residue leading to a dehydroalanine moiety. We report here the design, synthesis and radiosynthesis of a new fluoropyridine-based thiol [^18^F]FPySH and its conjugation via Michael addition on model dehydroalanine- or maleimide-containing biologics.

**Results:**

The preparation of cold reference and labeling precursor of [^18^F]FPySH was achieved and its radiosynthesis was fully automated, enabling production of the thiol prosthetic group with a 7 ± 2.1% radiochemical yield after two steps. The conjugation of [^18^F]FPySH to two model Dha-containing molecules was then carried out in reducing conditions, yielding the corresponding adducts in 30–45 min reaction time. Furthermore, [^18^F]FPySH was employed to radiolabel the maleimide-modified c(RGDfK) peptide, affording the radiofluorinated analogue in 15 min.

**Conclusion:**

We have developed an original [^18^F]-labeled thiol for site-selective conjugation and radiolabeling of Dha or maleimide-containing biomolecules of interest. Labeling of three model compounds was successfully carried out and gave the expected radiofluorinated adducts in less than 45 min, thus compatible with fluorine-18 half-life.

**Supplementary Information:**

The online version contains supplementary material available at 10.1186/s41181-022-00160-5.

## Background

Fluorine-18 labeled biologics such as peptides, proteins, polysaccharides or nucleic acids are valuable tools in molecular imaging by Positron Emission Tomography (PET) but their radiolabeling is challenging. Indeed, the drastic requirements (e.g. high temperature or non-aqueous conditions) usually crucial for radiofluorination are not compatible with the complexity and fragility of these compounds. To deal with these issues, strategies relying on two-steps methods involving the preparation of a ^18^F-labeled prosthetic group subsequently conjugated to the biomolecule of interest in biocompatible conditions have been developed (Kuhnast and Dollé 2010; Shuanglong et al. 2011; Krishnan et al. 2017). Amongst these prosthetic groups, some are amino selective labeling groups, like *N*-succinimidyl 4-[^18^F]fluorobenzoate ([^18^F]SFB) (Vaidyanathan and Zalutsky 2006), *N*-succinimidyl 3-(di-*tert*-butyl[^18^F]fluorosilyl)benzoate ([^18^F]SiFB) (Wängler et al. 2012b) or 6-[^18^F]fluoronicotinic acid tetrafluorophenyl ester ([^18^F]F-Py-TFP) (Olberg et al. 2010), displaying an activated ester for amide bond formation, or like 4-[^18^F]fluorobenzaldehyde ([^18^F]FBA) (Glaser et al. 2008) or *p*-(di-*tert*-butyl[^18^F]fluorosilyl) benzaldehyde ([^18^F]SiFA-A) (Schirrmacher et al. 2007), presenting and aldehyde function enabling oxime formation. Additionally, site selective radiolabeling methods based on the copper catalyzed alkyne–azide cycloaddition (CuAAC) have been developed (Rostovtsev et al. 2002; Tornøe et al. 2002). For instance, our group reported labeling reagents displaying an alkyne (2-[^18^F]fluoro-3-pent-4-yn-1-yloxypyridine, [^18^F]FPyKYNE) (Kuhnast et al. 2008) or an azido ([^18^F]FPyZIDE) (Roche et al. 2019) function for conjugation to peptides engineered with the corresponding azido or alkyne function.

**Fig. 1:**
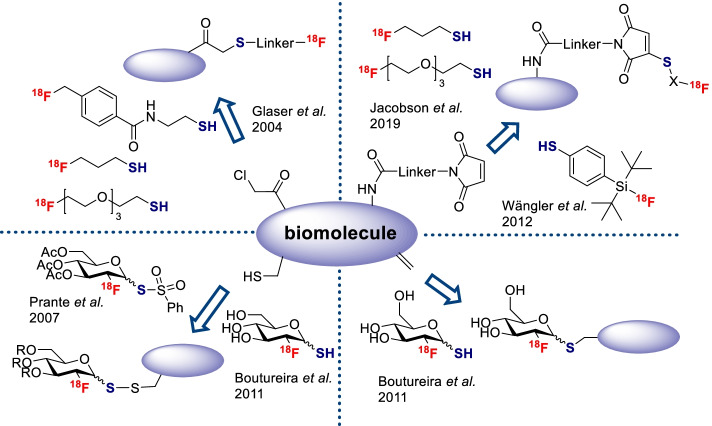
Examples of radiofluorinated thiol prosthetic groups

Michael addition of nucleophilic thiol moieties on α,β-unsaturated carbonyl entities has also been considerably employed for the labeling of biomolecules (Hoyle et al. 2010; Nair et al. 2014). Indeed, this reaction is robust, fast, biocompatible and produce highly stereospecific and regiospecific adducts, in accordance with the principles of click chemistry. Several thiol reactive compounds containing a maleimide function, such as *N*-{4-[(4-[^18^F]fluorobenzylidene)aminooxy]butyl}maleimide ([^18^F]FBAM) (Toyokuni et al. 2003), 1-[3-(2-[^18^F]fluoropyridin-3-yloxy)propyl]pyrrole-2,5-dione ([^18^F]FPyMe) (de Bruin et al. 2005) or *N*-[2-(4-[^18^F]fluorobenzamido)ethyl]maleimide ([^18^F]FBEM) (Cai et al. 2006), have been described. In 2020, Zhang et al. reported the synthesis of ^18^F-labeled vinyl sulfones and their use to radiolabel bioactive molecules and red blood cells in vivo (Zhang et al. 2020). However, there are few examples of radiofluorinated thiol prosthetic groups reported in the literature (Fig. 1).

In 2004, Glaser et al*.* reported the radiosynthesis of three [^18^F]fluorothiols and their use to radiolabel the chloroacetylated model peptide ClCH_2_C(O)-KGFGK-OH by nucleophilic substitution of chlorine (Glaser et al. 2004). However, radiolabeling of a chloroacetylated-RGD peptide with 3-[^18^F]fluoropropanethiol led to extensive decomposition due to the harsh conditions required for this reaction (Glaser et al. 2009). Lately, the Kiesewetter group employed two of these ^18^F-labeled thiols to radiolabel several maleimide-modified biomolecules of interest, such as a c(RGDfk)_2_ dimer, PSMA and tenascin-C or Sgc8 aptamers by Michael addition (Jacobson et al. 2019). Another example of ^18^F-labeled thiol was depicted by Schirrmacher and coworkers in 2012. They synthesised the silicon-fluoride acceptor reagent [^18^F]SiFA-SH by isotopic exchange and engaged it in a thiol-maleimide click chemistry reaction to radiolabel a maleimide-derivatized protein (Wängler et al. 2012a, b). Thiosugars, and more specifically thio-[^18^F]FDG, have also been used for the labeling of biomolecules. Prante et al*.* have thus described the three-step radiosynthesis of Ac_3_-[^18^F]FGlc-PTS, a phenylthiosulfonate derivative of [^18^F]FDG employed for the site-specific labeling of model peptide CAKAY and biologically relevant peptide c(RGDfC) via formation of a disulfide bond (Prante et al. 2007). They showed that the affinity of the [^18^F]-labeled peptide c(RGDfC(S,S’-Ac_3_-[^18^F]FGlc)) for αvβ3 integrin remained similar to its non-glycosylated analogue c(RGDfC). Likewise, the Davis group published a one-pot method for site-specific protein labeling involving direct thionation of [^18^F]FDG with Lawesson’s reagent followed by protein glycoconjugation via disulfide formation on a cysteine residue (Boutureira et al. 2011). Furthermore, this thiolated-[^18^F]FDG was successfully employed for 1,4 addition on a model protein containing a dehydroalanine (Dha) residue.

Dha has been extensively used in the recent years due to its efficient synthesis and its utility for site-specific peptide and protein modification in biocompatible conditions (Dadová et al. 2018). This α,β-unsaturated carbonyl residue, easily generated by selective modification of selenocysteine (Seebeck and Szostak 2006; Wang et al. 2007; Guo et al. 2008) or cysteine residues (Chalker et al. 2011; Bernardes et al. 2008), is a valuable tool for C-S (Seebeck and Szostak 2006; Wang et al. 2007; Guo et al. 2008; Nathani et al. 2013; Rowan et al. 2014), C-N (Freedy et al. 2017), C-Si (Vries and Roelfes 2020), C-Se (Reddy and Mugesh 2019; Oroz et al. 2021), C-P (He et al. 2019) and C-C (Wright et al. 2016; Yang et al. 2016; de Bruijn and Roelfes 2018; Josephson et al. 2020) bond formation or even cycloadditions on peptides or proteins (Bao et al. 2021). Regarding C-S bond formation, this technique has been implemented for post-translational modification of several proteins, including tau (Lindstedt et al. 2021), histones (Chalker et al. 2012), GFP (Nathani et al. 2013) or Aurora A kinase (Rowan et al. 2014).

**Fig. 2:**
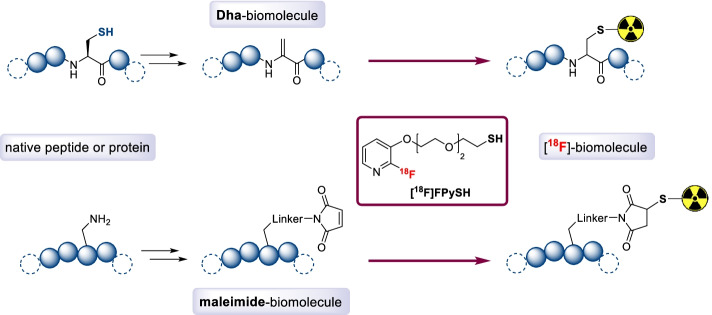
New fluorinated thiol prosthetic group: application to the labeling of Dha- and maleimide-modified peptides

In the present paper, we report the synthesis and radiosynthesis of an original thiol containing prosthetic group and its utilization to label several model compounds, from small molecules to more complex peptides, via a Michael addition reaction. Based on our previous experience with the radiolabeling of 2-fluoropyridines, we designed a 2-fluoropyridine presenting a thiol group on a PEG linker [^18^F]FPySH (Fig. 2). Looking into the available thiol reactive functions appropriate for biomolecule modification, we have prepared Dha- or maleimide-derivatized peptides and employed our new ^18^F-labeled thiol for their radiolabeling as a proof of concept.

## Results and discussion

### Synthesis of labeling precursor and cold references

**Scheme 1:**
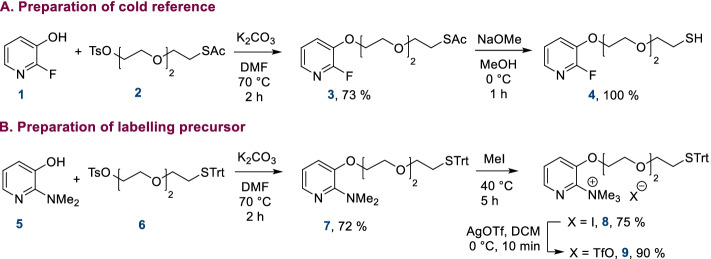
**A** preparation of cold reference **4**; **B** preparation of labeling precursor **9**

The first step was the preparation of cold reference **4** and corresponding labeling precursor **9** in two or three steps starting from commercially available pyridones (Scheme 1). The synthesis of **4** started by alkylation of 2-fluoro-3-pyridone **1** with the acetate-protected compound **2**. After reacting for 2 h in DMF at 70 °C in basic conditions, the expected protected thiopyridine **3** was obtained and treated with sodium methanolate to give cold reference **4** with a 73% yield over two steps. A similar approach was first envisioned for the preparation of an acetate-protected labeling precursor but the acetate protecting group proved unstable in radiofluorination conditions. Therefore, we opted for a trityl-protected labeling precursor. To this end, trityl-protected intermediate **6** was prepared and reacted with 2-dimethylamino-3-pyridone **5** at 70 °C in DMF to give **7**. Methylation of **7** was first attempted with methyl triflate but the trityl group was not stable in these conditions and dimerization of the compound was detected, even when the reaction was performed at 0 °C. To prevent this side-reaction, methylation of **7** was carried out by reaction with iodomethane at 50 °C, giving intermediate **8**. The methylation reaction was followed by anion exchange with silver triflate in DCM, leading to the expected labeling precursor **9** in an overall 49% yield in three steps.

**Scheme 2:**
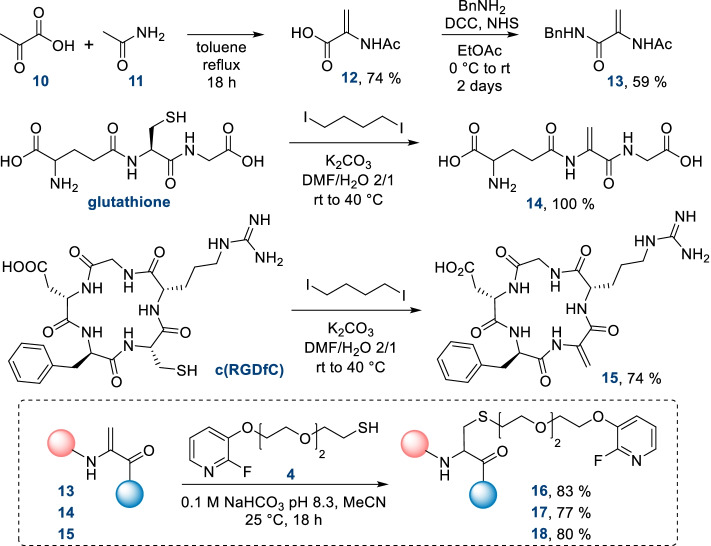
Preparation of model Dha-molecules **13**–**15** and their conjugation to fluorothiol **4**

Then, we implemented the preparation of Dha and maleimide biomolecules and their associated FPySH reference adducts. We carried out the synthesis of three Dha compounds and their conjugation to thiol reference **4** (Scheme 2). The preparation of the “small molecule” model **13** was achieved starting from pyruvic acid **10** and acetamide **11** according to described procedures (Dedeoğlu et al. 2013; Jukič et al. 2015). Unsaturated compound **12**, obtained after refluxing of **10** and **11** in toluene, was coupled with benzylamine to give the expected product **13** in two steps in a 44% yield. Dha-peptides **14** and **15** were prepared starting from glutathione and c(RGDfC), respectively, by action of 1,4-diiodobutane and potassium carbonate in a water/DMF mixture at 40 °C (Reddy and Mugesh 2019). With these Dha compounds in hand, we then turned to the preparation of their fluorothiol conjugates. Compound **4** was reacted overnight at room temperature with **13**, **14** and **15** in basic conditions, affording the corresponding reference adducts **16**, **17** and **18** in very good yields.

**Scheme 3:**
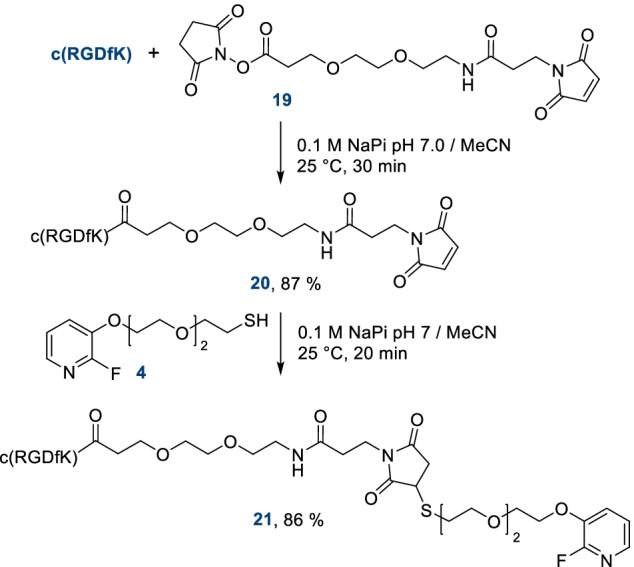
Preparation of c(RGDfK)-PEG-maleimide and its conjugation to fluorothiol **4**

In order to compare the reactivity of this new thiol prosthetic group towards different chemical moieties, we have also incorporated a maleimide on c(RGDfK) peptide and prepared its thio-fluoropyridine conjugate (Scheme 3). The amine of the lysine side chain of c(RGDfK) was first coupled to the commercially available *N*-hydrosuccinimide-PEG-maleimide **19**, affording c(RGDfK)-PEG-maleimide adduct **20** in 87% yield. Subsequent reaction with **4** in a 0.1 M sodium phosphate buffer/DMF mixture for 20 min led to the reference compound **21** with an 86% yield.

### Radiochemistry

**Scheme 4:**
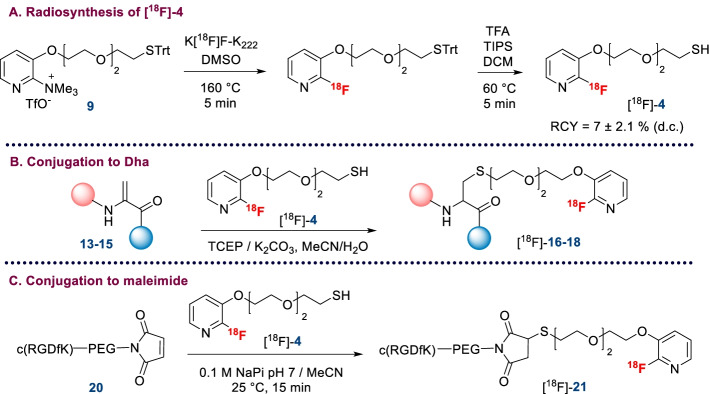
Labeling with [^18^F]FPySH. **A** Radiosynthesis of [^18^F]FPySH; **B** Addition of [^18^F]FPySH to Dha-compounds **13**–**15**; **C** Addition of [^18^F]FPySH to c(RGDfK)-PEG-maleimide **20**

**Fig. 3:**
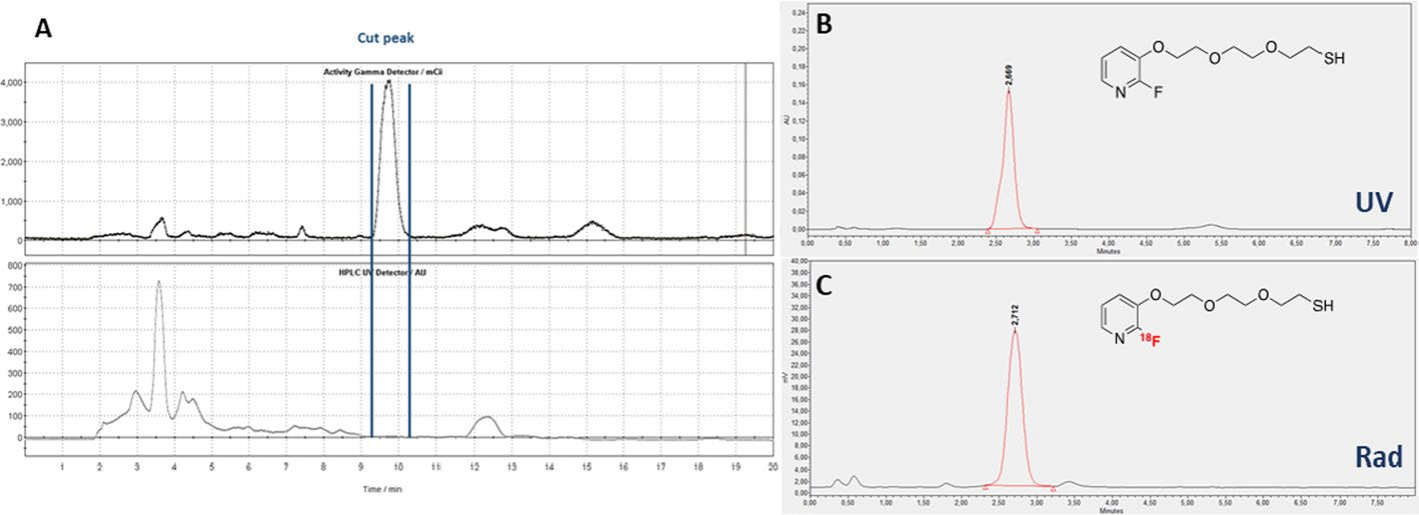
HPLC of [^18^F]FPysH radiosynthesis. **A** Preparative HPLC; **B** UV trace of reference compound **4**; **C** Radioactive trace of [^18^F]-**4 **after purification and formulation

[^18^F]FPySH ([^18^F]-**4**) was prepared by [^18^F]fluorine nucleophilic substitution of trimethylammonium precursor **9** (Scheme 4A). Following our previous work on fluoropyridines (Roche et al. 2019), we chose to work with a trimethylammonium precursor because the purification of the radiofluorinated compound is easier compared to the nitro precursor which has a polarity similar to its fluorinated analogue. Radiolabeling was carried out on a TRACERlab FXFn or FXNPro automate. Precursor **9** was reacted with the K[^18^F]F-K_222_ complex in DMSO at 160 °C for 5 min. The radiofluorinated trityl-protected prosthetic group was then purified on a Sep-Pak C18 cartridge and underwent deprotection by trifluoroacetic acid and triisopropylsilane treatment in dichloromethane at 60 °C. After evaporation of the solvent, the crude radiofluorinated thiol prosthetic group was purified by semi-preparative HPLC (*t*_R_ = 9–10 min, Fig. 3A). The pure [^18^F]-labeled product [^18^F]-**4** was obtained with a 7% ± 2.1 (n = 6) radiochemical yield (d.c.) after reformulation in acetonitrile on a Sep-Pak C18 cartridge after an average reaction time of 80 min. The chemical and radiochemical purities were higher than 95 and 98%, respectively, as attested by the quality control HPLC (Fig. 3B and C). Synthesis of [^18^F]FPySH was achieved with an average corrected molar activity of 101 ± 18 GBq/µmol. We have thus achieved the fully automated radiosynthesis of an original [^18^F]-labeled thiol in a more straightforward approach compared to the method developed by Boutureira et al., which requires the preparation of [^18^F]FDG followed by a time-consuming thionation reaction with Lawesson’s reagent (Boutureira et al. 2011).

**Fig. 4:**
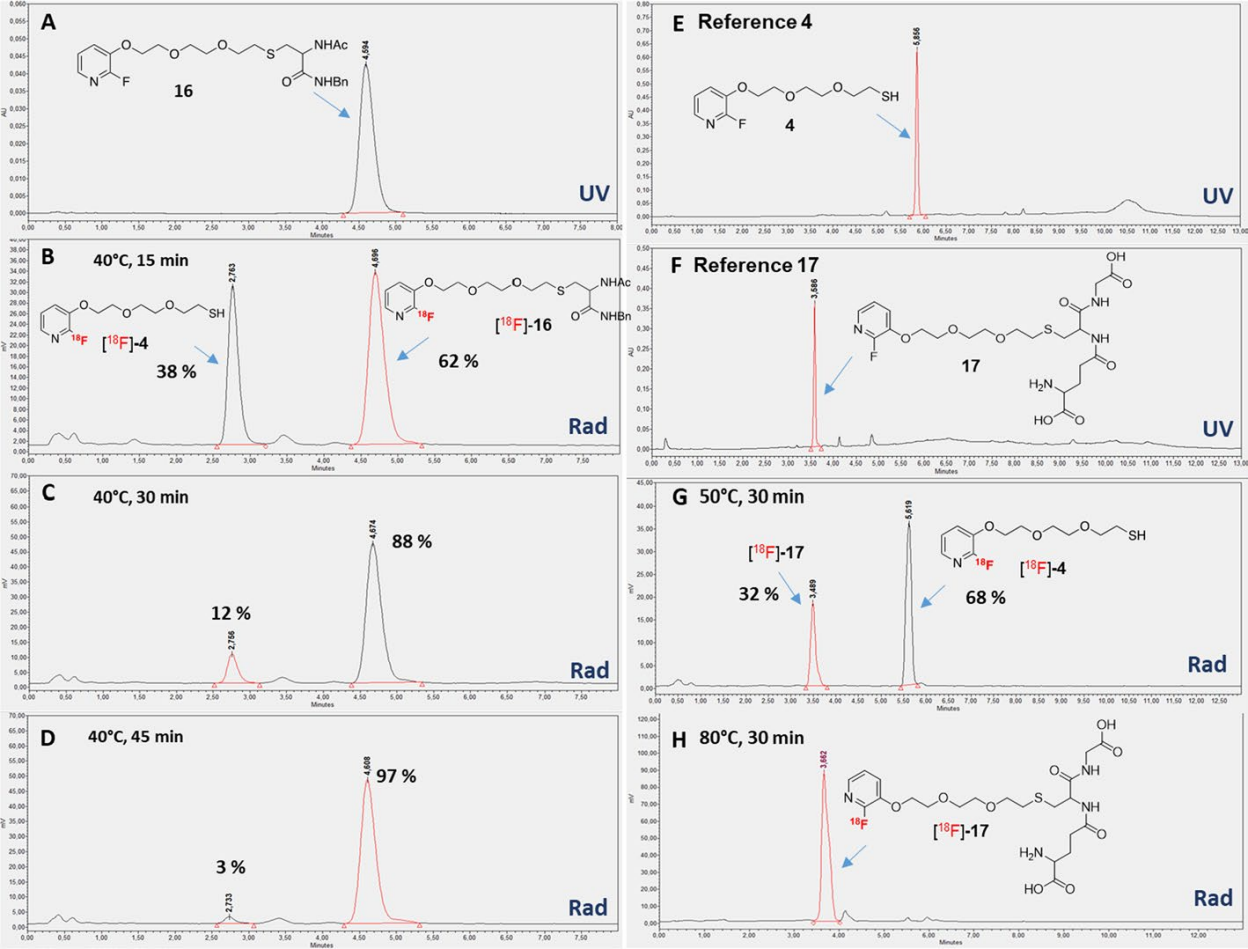
Analytic HPLC chromatograms of radiolabeling of **13** and **14** with [^18^F]FPySH. **A** UV trace of reference **16**; **B**–**D** RadioHPLC after 15, 30 & 45 min at 40 °C; **E** UV trace of reference **4**; **F** UV trace of reference **17**; **G** and **H** RadioHPLC traces after 30 min at 50 °C and 80 °C, respectively

With this radiolabeled compound in hand, we then examined the radiolabeling of the Dha- and maleimide-biomolecules with [^18^F]FPySH (Scheme 4B and C). Reaction was initially attempted on the model Dha “small molecule” **13** in MeCN at 25 °C without any reducing agent (Table 1, entry 1), but only dimerization of the thiol was observed in these conditions. Similar results were obtained by Glaser and co-workers in the case of the addition of ^18^F-labeled thiols on chloroacetylated peptides (Glaser et al. 2004). Tris(2-carboxyethyl)phosphine (TCEP, 8 mM) and potassium carbonate (8 mM) were therefore added to the reaction mixture, however conversion only reached 46% after 45 min when the addition was carried out at 25 °C (Table 1, entry 2), which compelled us to perform the reaction at 40 °C (Fig. 4A–D). At this temperature, 62% conversion was obtained after 15 min and near complete conversion of [^18^F]FPySH ([^18^F]-**4**) to [^18^F]-**16** was observed after 45 min (Table 1, entries 3 & 4). Only the expected adduct was detected by analytic HPLC with a radiochemical purity greater than 90%. For labeling of Dha-glutathione peptide **14**, the reaction was similarly achieved with TCEP (8 mM) and potassium carbonate (8 mM) in a MeCN/water 1/1 mixture (Fig. 4E–H). No conversion was observed at 25 °C, and up to 32% of [^18^F]-**17** was detected after 30 min at 50 °C (Table 1, entry 5). Incubation at 80 °C for 30 min enabled complete conversion of [^18^F]FPySH to [^18^F]-**17** with a radiochemical purity higher than 93% (Table 1, entry 6).

**Table 1:** Labeling of Dha and maleimide molecules with [^18^F]FPySH

Entry	Substrate^a^	Conditions^b^	Temperature (°C)	Reaction time (min)	Product	Conversion (%)
1	**13**	–	25	45	[^18^F]-**16**	0
2	**13**	TCEP, K_2_CO_3_	25	45	[^18^F]-**16**	46
3	**13**	TCEP, K_2_CO_3_	40	15	[^18^F]-**16**	62
4	**13**	TCEP, K_2_CO_3_	40	45	[^18^F]-**16**	97
5	**14**	TCEP, K_2_CO_3_	50	30	[^18^F]-**17**	32
6	**14**	TCEP, K_2_CO_3_	80	30	[^18^F]-**17**	100
7	**15**	TCEP, K_2_CO_3_	50	45	[^18^F]-**18**	0
8	**15**	TCEP, K_2_CO_3_	80	45	[^18^F]-**18**	0
9	**20**	TCEP, K_2_CO_3_	25	15	[^18^F]-**21**	> 95
10	**20**	–	25	15	[^18^F]-**21**	> 95

^a^All additions were carried out on 0.5–2.0 mg of substrate

^b^8 mM TCEP and 8 mM K_2_CO_3_ were used for entries 2–9

**Fig. 5:**
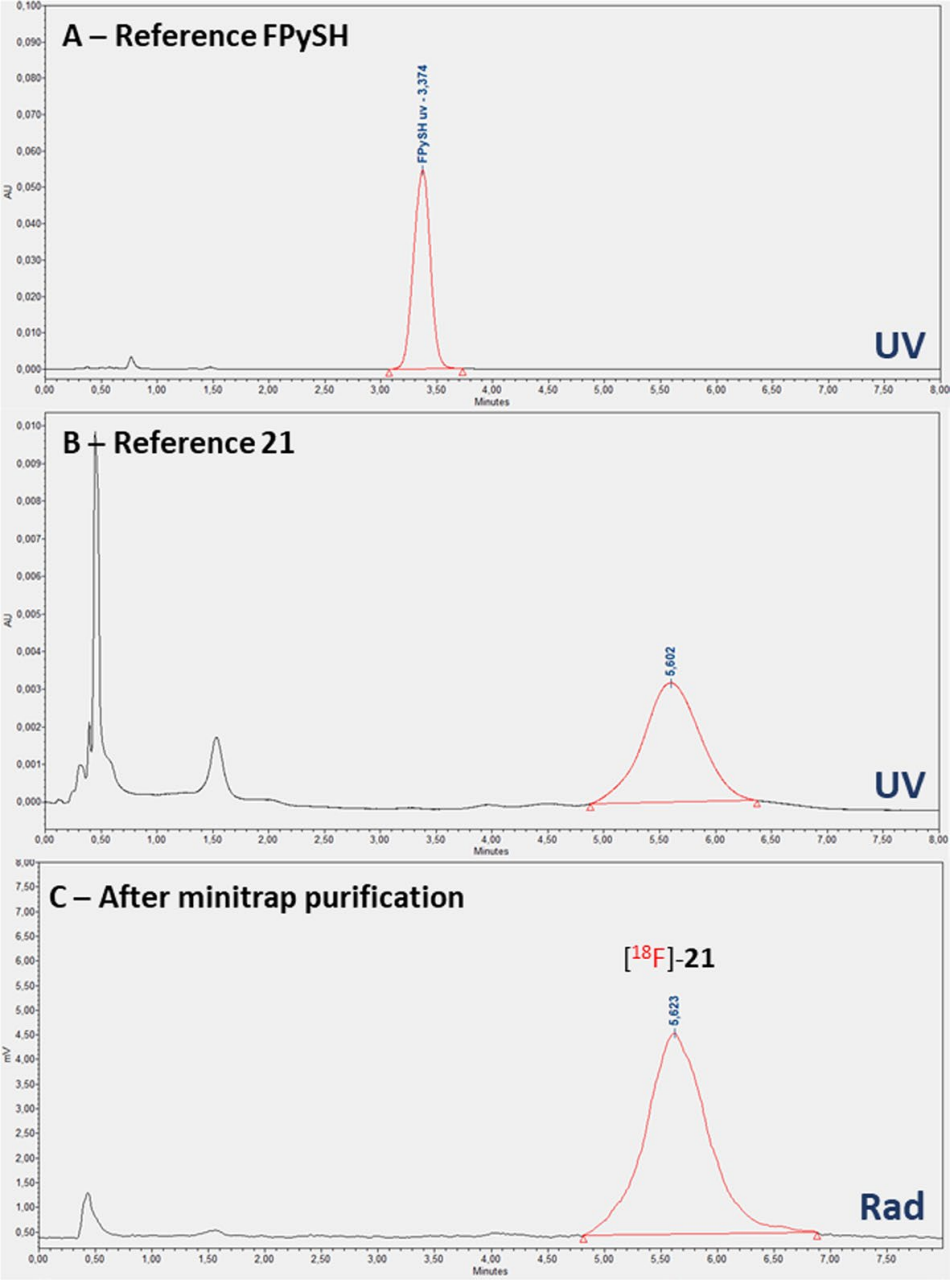
Analytic HPLC chromatograms of labeling of **20** with [^18^F]FPySH. **A** UV trace of reference FPySH; **B** UV trace of reference **21**; **C** RadioHPLC trace after minitrap G10 purification

We then turned our attention to the labeling of c(RGDf-Dha) peptide **15** with [^18^F]FPySH, however none of the investigated conditions led to the formation of the expected [^18^F]-**18** adduct (Table 1, entries 7 & 8). Reactions at lower temperatures were not efficient but higher temperatures combined to the basic reaction medium led to degradation of the peptide. Glaser et al*.* reported a comparable outcome when they attempted the labeling of a cyclic RGD peptide analogue in 2009, although the reaction was successful on other peptides (Glaser et al. 2009). This result suggests a limitation of our conjugation technique for the labeling of fragile biomolecules, even though the use of ^18^F-labeled thiol for the radiofluorination of model Dha-protein has been reported (Boutureira et al. 2011). Taking into account the results obtained by the Kiesewetter group for addition of the same ^18^F-labeled thiols on maleimide (Jacobson et al. 2019), we consequently experimented the labeling of c(RGDfK)-PEG-maleimide **20** with [^18^F]FPySH (Scheme 4C and Fig. 5). Incubation at 25 °C in 0.1 M sodium phosphate pH 7/MeCN 2/1 afforded the expected radiofluorinated conjugate [^18^F]-**21** with a 100% conversion after 15 min and a radiochemical purity above 96% (Table 1, entries 9 & 10). In this case, the addition of a reducing agent was not necessary and the reaction proceeded smoothly without TCEP. It should be noted that pH was adjusted to 7 with sodium hydrogen carbonate pH 8.3 when necessary.

## Conclusion

We have carried out the synthesis and radiosynthesis of [^18^F]-**4**, a novel radiofluorinated thiol for the labeling of biomolecules presenting a Dha or a maleimide moiety. This prosthetic group was then employed for the successful labeling of a model small molecule and a glutathione analogue modified with a Dha moiety. In all cases, labeling was complete in less than an hour and only one radiolabeled product was obtained. Although the addition of [^18^F]-**4** was not successful on the Dha-modified c(RGDfK), the reaction was completed in 15 min on maleimide-containing c(RGDfK), thus validating the use of our ^18^F-labeled thiol for the labeling of fragile peptides or proteins. We are currently working on the implementation of this technique for the labeling of more complex biomolecules like proteins.

## Methods

### Chemistry

For detailed information on chemistry methods and analysis techniques employed, see the “syntheses of precursors and cold references” section of the supporting information. Synthesis of prosthetic group reference and labeling precursor is described in Scheme 1, preparation of Dha- and maleimide-modified biomolecules and their conjugation to cold fluorothiol are depicted in Schemes 2 and 3.

### Radiochemistry

**Semi-preparative HPLC**: integrated to the TRACERLab FXFN/FXNPro (GE Medical Systems): S1122 Solvent Delivery System (Sykam); U.V. Detector K-2501 (Knauer); radioactivity γ detector; column Zorbax SB-C18, 5 µm, 9.4 × 250 mm (Agilent); H_2_O/MeCN/TFA: 65/35/0.1 (v/v/v); flow rate: 5 mL/min; detection λ = 254 nm.

**Analytic HPLC**: Waters Alliance 2690 equipped with a UV spectrophotometer (Photodiode Array Detector, Waters 996 (Waters)) and a Berthold LB509 radioactivity detector; column: analytical Symmetry-M^®^ C-18, 50 × 4.6 mm, 5 µm (Waters); solvent A: H_2_O containing Low-UV PIC^®^ B7 reagent (20 mL for 1000 mL), solvent B: H_2_O/CH_3_CN: 30/70 (v/v) containing Low-UV PIC^®^ B7 reagent (20 mL for 1000 mL), flow rate: 2.0 mL/min; U.V. detection at λ = 254 nm.

**Thin Layer Chromatography:** Thin Layer Chromatography (TLC) was performed on pre-coated plates of silica gel 60F254 (Merck) and eluted with ethyl acetate. Radioactive compounds were detected using a Mini-Scan and Flow-Count radioactive detection system (Bioscan) and Chromeleon software (Thermo Scientific).

**Radiosynthesis of [**^**18**^**F]-4:** No-carrier-added aqueous [^18^F]fluoride ion was produced via the [^18^O(p,n)^18^F] nuclear reaction by irradiation of a 2 mL [^18^O]water target (> 97%-enriched, CortecNet) on a Cyclone-18/9 cyclotron (18 MeV proton beam, IBA) and was transferred to the appropriate hot cell. Target hardware: commercial, 2-mL, two-port, stainless steel target holder equipped with a domed-end niobium cylinder insert. Target to hot cell liquid-transfer system: 60 m PTFE line (0.8 mm internal diameter; 1/16 inch external diameter), 2.0 bar helium drive pressure, transfer time 3–6 min. Typical production of [^18^F]fluoride ion at the end of bombardment for a 25 µA, 30 min irradiation: 27–30 GBq. The aqueous solution containing [^18^F]fluoride anions was automatically transferred to the TRACERLab FX-FN or FX N Pro after the end of irradiation. The irradiated water was then sucked through an anion exchange cartridge (Sep-Pak^®^ Accell Plus QMA Plus Light cartridge, Waters) to fix [^18^F]fluoride anions and remove the enriched water. The [^18^F]fluoride anions were eluted from the resin and transferred to the reactor with a K_2_CO_3_/K_222_ solution (1 mL of water/acetonitrile 30/70 containing 4.5 mg of K_2_CO_3_ and 12 to 15 mg of Kryptofix^®^ 222). Finally, the K[^18^F]F-K_222_ complex was prepared by evaporation of the solution in two heating steps: (i) 60 °C for 7 min under reduced pressure along with a stream of helium and (ii) 120 °C for 5 min under vacuum. After cooling to 35 °C, radiofluorination was carried out by addition of the labeling precursor **9** (5 mg) in solution in dimethyl sulfoxide (0.7 mL) to the standard activated K[^18^F]F-K_222_ complex and heating the resulting mixture at 160 °C for 5 min. After cooling to 40 °C, the crude was diluted with 8 mL H_2_O and pre-purified through a C18 cartridge (Sep-Pak^®^Plus C18 cartridge, Waters). The trityl-protected [^18^F]FPySH was eluted from the C18 cartridge and transferred back to the reactor with 3 mL DCM. Trityl protecting group was removed by addition of a mixture of TIPS (120 µL) and TFA (160 µL) in DCM (1 mL) and heating for 5 min at 60 °C. After cooling down to 40 °C, DCM was removed by evaporation in two heating steps: (i) 70 °C for 5 min with a stream of helium and (ii) 90 °C for 2 min under vacuum. The crude was treated with a solution of TCEP (10 mg in H_2_O/MeCN/DMSO 3/1/1) for 5 min at 50 °C before HPLC injection (Zorbax, H_2_O/MeCN 65/35 + 0.1% TFA, 5 mL/min). The retention time of [^18^F]FPySH is around 10 min (Fig. 3A). The final formulation was performed automatically using a Sep-Pak^®^Plus C18 cartridge (Waters) and the purified prosthetic reagent was recovered after elution of the C18 cartridge with MeCN (2 mL). Chemical and radiochemical purities were assessed after reducing step by analytical HPLC (A/B: 65/35 *t*_R_ = 2.71 min for [^18^F]-**4**, *t*_R_ = 2.67 min for **4**, Fig. 3B-C). 1.52–1.85 GBq of the expected [^18^F]FPySH were obtained after ~ 80 min (RCY: 7 ± 2.1% d.c., n = 6). Molar activity of the radiotracer was calculated from three consecutive HPLC analyses (average) and determined as follows: the area of the UV absorbance peak corresponding to the radiolabeled product was measured (integrated) on the HPLC‐chromatogram and compared with a standard curve relating mass to UV absorbance. The average decay corrected molar activity was 101 ± 18 GBq/μmol (n = 6).

**Conjugation of [**^**18**^**F]-4 to Dha-containing biomolecules 13** and **14.** [^18^F]-**4** (200 µL of MeCN solution, ~ 150 MBq), TCEP (20 µL of 0.1 M solution in water) and K_2_CO_3_ (20 µL of a 0.1 M solution in water) were added to 0.5–2 mg of Dha-molecule **13** (neat) or **14** (in 200 µL H_2_O) and the reaction mixture was incubated at 40–80 °C. Reaction was monitored by radioHPLC (A/B: 65/35 for addition on **13**, gradient method for **14**).

**Conjugation of [**^**18**^**F]-4 to maleimide-containing biomolecule 20.** [^18^F]-**4** (200 µL of MeCN solution, ~ 150 MBq) were added to a solution of **20** in 100 µL 0.1 M NaPi pH 7. The pH of the mixture was adjusted to ~ 7 by adding 0.1 M NaHCO_3_ pH 8.3 (20 µL) and the reaction mixture was incubated at room temperature. Reaction was monitored by radioHPLC and radio-TLC. Purification by Minitrap G10 afforded the purified [^18^F]-**21** with a 72% radiochemical yield (d.c.).

## Supplementary Information

**Additional file 1**. Details for the syntheses of precursors and references and NMR spectra

## Data Availability

The data associated to this research work are available in this manuscript or in the online supplementary file.

## Abbreviations

Am: Molar activity
DCM: Dichloromethane
Dha: Dehydroalanine
DMF: Dimethylformamide
DMSO: Dimethyl sulfoxide
HPLC: High pressure liquid chromatography
MeCN: Acetonitrile
MeOH: Methanol
NaPi: Sodium phosphate
n.d.c.: Non-decay-corrected
PEG: Polyethylene glycol
PET: Positron emission tomography
RCY: Radiochemical yield
r.t.: Room temperature
TFA: Trifluoroacetic acid
TIPS: Triisopropyl silane
