# Supplementary material for: Novel [18F]-labeled thiol for the labeling of Dha- or maleimide-containing biomolecules
Source: EJNMMI Radiopharm Chem. 2022 Apr 6;7:7. doi: 10.1186/s41181-022-00160-5 (PMC8986957; doi:10.1186/s41181-022-00160-5)
Supplement: Supplementary file 1 — Additional file 1. Details for the syntheses of precursors and references and NMR spectra [file 41181_2022_160_MOESM1_ESM.docx]

**Novel [^18^F]-labeled thiol for the labeling of Dha- or maleimide-containing biomolecules**

Mylène Richard, Françoise Hinnen and Bertrand Kuhnast

**Supplementary Information**

Table of contents

I. Syntheses of precursors and cold references 3

II. ^1^H and ^13^C NMR spectra 13

III. Bibliography 27

# I. Syntheses of precursors and cold references

**Materials and methods**

All commercially available reagents and solvents were purchased from Sigma-Aldrich (France) and used without further purification. Purifications of products were carried out by column chromatography using Merck silica gel (63-200 µm). ^1^H NMR (400 MHz) and ^13^C NMR (100 MHz) were measured on a Brucker Avance 400 MHz spectrometer. Chemical shifts are reported in parts per million (ppm, δ) downfield from residual solvents peaks and coupling constants are reported in Hertz (Hz). Splitting patterns are designated as singlet (s), broad singlet (br. s), doublet (d), triplet (t), quartet (q) and quintet (quin). Splitting patterns that could not be interpreted or easily visualized are designated as multiplet (m). High-resolution mass spectra (HRMS) were performed by the "Fédération de Recherche" ICOA/CBM (FR2708) platform on a Bruker maXis mass spectrometer coupled to a Dionex Ultimate 3000 HPLC system (Thermo). Melting points (Mp) were measured on an electrothermal IA9200 and are reported in °C.

**2-(2-(2-(Acetylthio)ethoxy)ethoxy)ethyl 4-methylbenzenesulfonate 2**

Potassium thioacetate (1.14 g, 10 mmol, 2 eq.) was added to a solution of 2-[2-(2-chloroethoxy)-ethoxy]ethanol (730 µL, 5 mmol, 1 eq) in DMF (7 mL) under argon and the reaction mixture was stirred at room temperature until completion. Then, EtOAc (30 mL) was added to the reaction mixture and the organic layer was washed with 5% LiCl (3 x 10 mL), dried over anhydrous Na_2_SO_4_, and concentrated under reduced pressure. The residue was purified by flash chromatography (n-heptane/EtOAc: 5/5 to 100 % EtOAc) to give 830 mg of 2-(2-(2-(acetylthio)ethoxy)ethoxy)ethanol (72 % yield, yellow oil).

Triethylamine (1.05 mL, 7.5 mmoles, 2.5 eq.), tosyl chloride (1.14 g, 6 mmoles, 2 eq.) and DMAP (36 mg, 0.3 mmoles, 0.1 eq.) were added to a solution of 2-(2-(2-(acetylthio)ethoxy)ethoxy)ethanol (624 mg, 3 mmoles, 1 eq.) in 20 mL dry CH_2_Cl_2_. The mixture was stirred at room temperature until completion (5 hours). CH_2_Cl_2_ (20 mL) was then added and the solution was washed with 1 M HCl (15 mL), saturated aqueous NaHCO_3_ (15 mL) and brine (15 mL). The organic layer was dried over anhydrous Na_2_SO_4_ and concentrated under reduced pressure. The residue was purified by flash chromatography (n-heptane/EtOAc: 8/2) to give 900 mg of **2** (83 % yield, colorless oil). **^1^H (CDCl_3_, 400 MHz):** 7.79 (app d, 2H, *J* = 8.4 Hz, 2 *H*-Ar), 7.34 (app d, 2H, *J* = 8.4 Hz, 2 *H*-Ar), 4.16-4.14 (m, 2H, C*H*_2_), 3.69-3.67 (m, 2H, C*H*_2_), 3.58-3.52 (m, 6H, 3 C*H*_2_), 3.05 (t, 2H, *J* = 6.5 Hz, C*H*_2_-S), 2.44 (s, 3H, C*H*_3_), 2.33 (s, 3H, C*H*_3_). **^13^C (CDCl_3_, 100 MHz):** 195.6 (*C*=O), 144.8 (*C*_quat_-Ar), 133.0 (*C*_quat_-Ar), 129.8 (2 *C*-Ar), 128.0 (2 *C*-Ar), 70.7 (*C*H_2_), 70.3 (*C*H_2_), 69.8 (*C*H_2_), 69.3 (*C*H_2_), 68.8 (*C*H_2_-OTs), 30.6 (*C*H_3_), 28.8 (*C*H_2_-S), 21.7 (*C*H_3_). **HRMS (ESI):** m/z calcd for C_15_H_23_O_6_S_2_ [M+H]^+^: 298.1310; found: 298.1313.

**3-(2-(2-(2-(Acetylthio)ethoxy)ethoxy)ethoxy)-2-fluoropyridine 3**

K_2_CO_3_ (276 mg, 2 mmol, 2 eq) was added to a solution of 2-fluoro-3-pyridone **1** (170 mg, 1.5 mmol, 1.5 eq) in dry DMF (10 mL) and the resulting solution was stirred at room temperature for 30 minutes. A solution of **2** (362 mg, 1 mmol, 1 eq) in dry DMF (2 mL) was added and the reaction mixture was stirred at 70 °C until completion (~2 hours). After cooling to room temperature, EtOAc was added (30 mL) and the organic layer was washed with 5 % LiCl, 1 M NaOH and H_2_O, dried over anhydrous Na_2_SO_4_ and concentrated under reduced pressure. The residue was purified by flash chromatography (n-heptane/EtOAc: 7/3 to 5/5) to give **3** as a colorless gum (222 mg, 73 % yield). **^1^H (CDCl_3_, 400 MHz):** 7.75 (ddd, 1H, *J*_5,6_ = 4.8, *J*_4,6_ = 1.6, *J*_H-F_ = 1.6 Hz, *H*-6), 7.33 (ddd, 1H, *J*_H-F_ = 10.3, *J*_4,5_ = 7.9, *J*_4,6_ = 1.6 Hz, *H*-4), 7.10 (ddd, 1H, *J*_4,5_ = 7.9, *J*_5,6_ = 4.8, *J*_H-F_ = 0.5 Hz, *H*-5), 4.21 (dd, 2H, *J* = 5.0, *J* = 4.5 Hz, C*H*_2_), 3.90-3.88 (m, 2H, C*H*_2_), 3.73-3.71 (m, 2H, C*H*_2_), 3.65-3.62 (m, 2H, C*H*_2_), 3.60 (t, 2H, *J* = 6.5 Hz, C*H*_2_), 3.09 (t, 2H, *J* = 6.4 Hz, C*H*_2_-S), 2.33 (s, 3H, C*H*_3_). **^13^C (CDCl_3_, 100 MHz):** 195.6 (*C*=O), 153.9 (d, ^1^*J*_C-F_ = 234.6 Hz, *C*_2_-Ar), 142.2 (d, ^2^*J*_C-F_ = 25.6 Hz, *C*_3_-Ar), 137.7 (d, ^3^*J*_C-F_ = 13.1 Hz, *C*_6_-Ar), 123.5 (d, ^3^*J*_C-F_ = 4.4 Hz, *C*_4_-Ar), 121.7 (d, ^4^*J*_C-F_ = 4.3 Hz, *C*_5_-Ar), 70.9 (*C*H_2_), 70.4 (*C*H_2_), 69.8 (*C*H_2_), 69.6 (*C*H_2_), 69.1 (*C*H_2_), 30.6 (*C*H_3_), 28.8 (*C*H_2_-S). **HRMS (ESI):** m/z calcd for C_13_H_19_FNO_4_S [M+H]^+^: 304.1013; found: 304.1015.

**3-(2-(2-(2-Thioethoxy)ethoxy)ethoxy)-2-fluoropyridine 4**

To a solution of **3** (151 mg, 0.5 mmoles, 1 eq) in dry MeOH (5 mL) was added MeONa (0.3 mL) dropwise at 0°C under argon. After stirring for 1 hour at 0°C, 1 M HCl was added until pH reached 6-7 and the product was extracted with EtOAc (3 x 20 mL). The organic layer was dried over anhydrous Na_2_SO_4_ and concentrated under reduced pressure. **4** was used without further purification (colorless oil, 130 mg, quantitative yield). **^1^H (CDCl_3_, 400 MHz):** 7.74 (td, 1H, *J*_5,6_ = 4.9, *J*_4,6_ = 1.7, *J*_H-F_ = 1.7 Hz, *H*-6), 7.32 (ddd, 1H, *J*_H-F_ = 10.2, *J*_4,5_ = 7.9, *J*_4,6_ = 1.7 Hz, *H*-4), 7.09 (ddd, 1H, *J*_4,5_ = 7.9, *J*_5,6_ = 4.9, *J*_H-F_ = 0.8 Hz, *H*-5), 4.21 (dd, 2H, *J* = 5.4, *J* = 4.0 Hz, C*H*_2_), 3.90-3.87 (m, 2H, C*H*_2_), 3.73-3.71 (m, 2H, C*H*_2_), 3.65-3.62 (m, 2H, C*H*_2_), 3.61 (t, 2H, *J* = 6.5 Hz, C*H*_2_), 2.68 (td, 2H, *J* = 8.2 Hz, *J* = 6.4 Hz, C*H*_2_-S), 1.57 (t, 1H, *J* = 8.2 Hz, S*H*). **^13^C (CDCl_3_, 100 MHz):** 153.9 (d, ^1^*J*_C-F_ = 241.3 Hz, *C*_2_-Ar), 142.2 (d, ^2^*J*_C-F_ = 25.8 Hz, *C*_3_-Ar), 137.7 (d, ^3^*J*_C-F_ = 13.3 Hz, *C*_6_-Ar), 123.4 (d, ^3^*J*_C-F_ = 4.2 Hz, *C*_4_-Ar), 121.7 (d, ^4^*J*_C-F_ = 4.1 Hz, *C*_5_-Ar), 72.9 (*C*H_2_), 70.9 (*C*H_2_), 70.3 (*C*H_2_), 69.5 (*C*H_2_), 69.1 (*C*H_2_), 24.3 (*C*H_2_-S). **HRMS (ESI):** m/z calcd for C_11_H_17_FNO_3_S [M+H]^+^: 262.0908; found: 262.0913.

**2-(2-(2-(Tritylthio)ethoxy)ethoxy)ethyl 4-methylbenzenesulfonate 6**

To a solution of NaH (60 %, 100 mg, 4 mmoles, 1 eq) in dry DMF (10 mL) was added triphenylmethanethiol (1.1 g, 4 mmol, 1 eq.) portion wise at 0 °C under argon. After stirring for 30 minutes at 0 °C, a solution of 2-[2-(2-chloroethoxy)-ethoxy]ethanol (580 µL, 4 mmol, 1eq) in DMF (5 mL) was added dropwise at 0 °C. The ice bath was removed and the resulting solution was stirred at room temperature until completion. EtOAc (30 mL) was added to the reaction mixture and the organic layer was washed with 5 % LiCl (3 x 10 mL), dried over anhydrous Na_2_SO_4_, and concentrated under reduced pressure. The residue was used without further purification. The residue was taken up in dry CH_2_Cl_2_ (40 mL) and triethylamine (1.4 mL, 10 mmoles, 2.5 eq.), tosyl chloride (1.52 g, 8 mmoles, 2 eq.) and DMAP (49 mg, 0.4 mmoles, 0.1 eq.) were added under argon. The mixture was stirred at room temperature until completion (5 hours). CH_2_Cl_2_ (40 mL) was then added and the solution was washed with 1 M HCl (20 mL), saturated aqueous NaHCO_3_ (20 mL) and brine (20 mL). The organic layer was dried over anhydrous Na_2_SO_4_ and concentrated under reduced pressure. The residue was purified by flash chromatography (n-heptane/EtOAc: 8/2 to 6/4) to give 1.8 g of **6** (80 % yield, colorless oil). **^1^H (CDCl_3_, 400 MHz):** 7.71-7.68 (m, 2H, 2 *H*-Ar), 7.34-7.31 (m, 6H, 6 *H*-Ar), 7.24-7.17 (m, 8H, 8 *H*-Ar), 7.14-7.10 (m, 3H, 3 *H*-Ar), 4.05 (t, 2H, *J* = 4.9 Hz, C*H*_2_), 3.57 (t, 2H, *J* = 4.9 Hz, C*H*_2_), 3.42-3.40 (m, 2H, C*H*_2_), 3.31-3.28 (m, 2H, C*H*_2_), 3.18 (t, 2H, *J* = 6.9 Hz, C*H*_2_), 2.34 (s, 3H, C*H*_3_), 2.33 (t, 2H, *J* = 6.9 Hz, C*H*_2_). **^13^C (CDCl_3_, 100 MHz):** 144.8 (4 *C*_quat_-Ar), 133.0 (*C*_quat_-Ar), 129.8 (2 *C*-Ar), 129.6 (6 *C*-Ar), 128.0 (2 *C*-Ar), 127.9 (6 *C*-Ar), 126.7 (3 *C*-Ar), 70.6 (*C*H_2_), 70.1 (*C*H_2_), 69.7 (*C*H_2_), 69.3 (*C*H_2_), 68.7 (*C*H_2_), 66.6 (*C*Ph_3_), 31.9, 31.7 (*C*H_3_, *C*H_2_-S), 21.7 (*C*H_3_). **LCMS (ESI):** m/z calcd for C_32_H_34_O_5_S_2_Na [M+Na]^+^: 585.2; found: 585.3.

***N,N*-Dimethyl-3-(2-(2-(2-(tritylthio)ethoxy)ethoxy)ethoxy)-pyridin-2-amine 7**

K_2_CO_3_ (276 mg, 2 mmol, 2 eq) was added to a solution of 2-dimethylamino-3-pyridone **5** (207 mg, 1.5 mmol, 1.5 eq) in dry DMF (10 mL) and the resulting solution was stirred at room temperature for 30 minutes. A solution of **6** (562 mg, 1 mmol, 1 eq) in dry DMF (2 mL) was added and the reaction mixture was stirred at 70 °C until completion (~2 hours). After cooling to room temperature, EtOAc (30 mL) was added and the organic layer was washed with 5 % LiCl, 1 M NaOH and H_2_O, dried over anhydrous Na_2_SO_4_ and concentrated under reduced pressure. The residue was purified by flash chromatography (n-heptane/EtOAc: 7/3) to yield **7** as a colorless gum (380 mg, 72 % yield). **^1^H (CDCl_3_, 400 MHz):** 7.86 (dd, 1H, *J*_5,6_ = 4.8, *J*_4,6_ = 1.5, *H*-6), 7.44-7.41 (m, 6H, 6 *H*-Ar), 7.31-7.26 (m, 6H, 6 *H*-Ar), 7.23-7.19 (m, 3H, 3 *H*-Ar), 7.00 (dd, 1H, *J*_4,5_ = 7.8, *J*_4,6_ = 1.5 Hz, *H*-4), 6.71 (dd, 1H, *J*_4,5_ = 7.8, *J*_5,6_ = 4.8 Hz, *H*-5), 4.11 (dd, 2H, *J* = 5.9, *J* = 4.9 Hz, C*H*_2_), 3.86 (dd, 2H, *J* = 5.9, *J* = 4.9 Hz, C*H*_2_), 3.64 (dd, 2H, *J* = 6.1, *J* = 3.4 Hz, C*H*_2_), 3.48 (dd, 2H, *J* = 6.1, *J* = 3.4 Hz, C*H*_2_), 3.33 (t, 2H, *J* = 6.9 Hz, C*H*_2_), 2.99 (s, 6H, 2 C*H*_3_), 2.44 (t, 2H, *J* = 6.9 Hz, C*H*_2_-S). **^13^C (CDCl_3_, 100 MHz):** 153.2 (*C*_2_-Ar), 144.5 (*C*_3_-Ar), 144.8 (3 *C*-Ar_quat_), 139.1 (*C*_6_-Ar), 129.6 (6 *C*-Ar), 127.9 (6 *C*-Ar), 126.7 (3 *C*-Ar), 119.1 (*C*_4_-Ar), 115.4 (*C*_5_-Ar), 70.7 (*C*H_2_), 70.3 (*C*H_2_), 69.7 (*C*H_2_), 69.6 (*C*H_2_), 67.7 (*C*H_2_), 66.6 (*C*Ph_3_), 40.9 (2 *C*H_3_), 31.7 (*C*H_2_-S). **HRMS (ESI):** m/z calcd for C_32_H_37_N_2_O_3_S [M+H]^+^: 529.2519; found: 529.2523.

***N,N,N*-Trimethyl-3-(2-(2-(2-(tritylthio)ethoxy)ethoxy)ethoxy)-pyridin-2-aminium iodide 8**

A solution of **7** (53 mg, 0.1 mmol) in iodomethane (1.0 mL) was stirred at 40 °C for 5 hrs. The reaction mixture was diluted with Et_2_O (5 mL), triturated and the resulting precipitate was filtered and washed with Et_2_O. The product was purified by flash chromatography (CH_2_Cl_2_/MeOH: 95/5) to give **8** (50 mg, 75%, yellow sticky gum). **^1^H (DMSO-d_6_, 400 MHz):** 8.18 (dd, 1H, *J*_5,6_ = 4.6, *J*_4,6_ = 1.3 Hz, *H*-6), 7.96 (dd, 1H, *J*_4,5_ = 8.4, *J*_4,6_ = 1.3 Hz, *H*-4), 7.73 (dd, 1H, *J*_4,5_ = 8.4, *J*_5,6_ = 4.6 Hz, *H*-5), 7.36-7.30 (m, 12H, 12 *H*-Ar), 7.27-7.23 (m, 3H, 3 *H*-Ar), 4.42 (dd, 2H, *J* = 5.9, *J* = 2.8 Hz, C*H*_2_), 3.84 (dd, 2H, *J* = 5.9, *J* = 2.8 Hz, C*H*_2_), 3.59 (s, 9H, 3 C*H*_3_), 3.53 (dd, 2H, *J* = 6.1, *J* = 3.2 Hz, C*H*_2_), 3.36 (dd, 2H, *J* = 6.1, *J* = 3.2 Hz, C*H*_2_), 3.21 (t, 2H, *J* = 6.6 Hz, C*H*_2_), 2.27 (t, 2H, *J* = 6.6 Hz, C*H*_2_-S). **^13^C (DMSO-d_6_, 100 MHz):** 147.0 (*C*_2_-Ar), 144.4 (3 *C*-Ar_quat_), 142.9 (*C*_3_-Ar), 138.5 (*C*_6_-Ar), 129.1 (6 *C*-Ar), 128.3 (*C*_5_-Ar), 128.0 (6 *C*-Ar), 126.7 (3 *C*-Ar), 125.5 (*C*_4_-Ar), 69.5 (*C*H_2_), 69.4 (*C*H_2_), 68.9 (*C*H_2_), 68.6 (*C*H_2_), 68.0 (*C*H_2_), 66.0 (*C*Ph_3_), 53.3 (3 *C*H_3_), 31.3 (*C*H_2_-S). **HRMS (ESI):** m/z calcd for C_33_H_39_N_2_O_3_S [M]^+^: 543.2676; found: 543.2676.

***N,N,N*-Trimethyl-3-(2-(2-(2-(tritylthio)ethoxy)ethoxy)ethoxy)-pyridin-2-aminium trifluoromethane sulfonate 9**

AgOTf (42 mg, 0.16 mmol, 1.05 eq) was added to a solution of **8** (104 mg, 0.15 mmol, 1 eq) in dry DCM (3.0 mL) at 0 °C. After stirring for 20 minutes at 0 °C, the solvent was evaporated and the product was purified by flash chromatography (CH_2_Cl_2_/MeOH: 95/5 to 90/10) to give **9** (100 mg, 93 %, yellow gum). **^1^H (DMSO-d_6_, 400 MHz):** 8.17 (dd, 1H, *J*_5,6_ = 4.6, *J*_4,6_ = 1.3 Hz, *H*-6), 7.95 (dd, 1H, *J*_4,5_ = 8.4, *J*_4,6_ = 1.3 Hz, *H*-4), 7.72 (dd, 1H, *J*_4,5_ = 8.4, *J*_5,6_ = 4.6 Hz, *H*-5), 7.36-7.29 (m, 12H, 12 *H*-Ar), 7.27-7.23 (m, 3H, 3 *H*-Ar), 4.41 (dd, 2H, *J* = 5.9, *J* = 2.8 Hz, C*H*_2_), 3.83 (dd, 2H, *J* = 5.9, *J* = 2.8 Hz, C*H*_2_), 3.58 (s, 9H, 3 C*H*_3_), 3.53 (dd, 2H, *J* = 6.1, *J* = 3.2 Hz, C*H*_2_), 3.36 (dd, 2H, *J* = 6.1, *J* = 3.2 Hz, C*H*_2_), 3.21 (t, 2H, *J* = 6.6 Hz, C*H*_2_), 2.27 (t, 2H, *J* = 6.6 Hz, C*H*_2_-S). **^13^C (DMSO-d_6_, 100 MHz):** 147.0 (*C*_2_-Ar), 144.4 (3 *C*-Ar_quat_), 142.9 (*C*_3_-Ar), 138.5 (*C*_6_-Ar), 129.1 (6 *C*-Ar), 128.3 (*C*_5_-Ar), 128.0 (6 *C*-Ar), 126.7 (3 *C*-Ar), 125.5 (*C*_4_-Ar), 69.5 (*C*H_2_), 69.4 (*C*H_2_), 68.9 (*C*H_2_), 68.6 (*C*H_2_), 68.0 (*C*H_2_), 66.1 (*C*Ph_3_), 53.3 (3 *C*H_3_), 31.3 (*C*H_2_-S). **HRMS (ESI):** m/z calcd for C_33_H_39_N_2_O_3_S [M]^+^: 543.2676; found: 543.2687.

**2-Acetamidoacrylic acid 12**

Prepared according to a literature report with some modifications (Dedeoğlu et al. 2013). Pyruvic acid (5.83 ml, 0.084 mol, 2.1 eq) and acetamide (2.36 g, 0.04 mol, 1 eq) were added to 150 ml toluene in a round bottomed flask fitted with a Dean-Stark apparatus and a reflux condenser. The solution was refluxed overnight. Then, half of the toluene was removed under reduced pressure and a precipitate was formed upon cooling at room temperature. This solid was filtered, washed with toluene and recrystallized in MeOH to give **12** as a white solid (3.8 g, 74 % yield). **^1^H (DMSO-d_6_, 400 MHz):** 13.27 (br s, 1H, CO_2_*H*), 9.11 (br s, 1H, N*H*), 6.25 (s, 1H, C=C*H*_2_), 5.67 (s, 1H, C=C*H*_2_), 2.03 (s, 3H, C*H*_3_). **^13^C (DMSO-d_6_, 100 MHz):** 169.3 (*C*=O), 165.0 (*C*=O), 133.2 (*C*=CH_2_), 107.7 (C=*C*H_2_), 23.7 (*C*H_3_).

**2-Acetamido-*N*-benzylacrylamide 13**

Prepared according to a previously reported procedure (Jukič et al. 2015). *N*-Hydroxysuccinimide (253 mg, 2.2 mmol, 1.1 eq.) and *N,N'*-dicyclohexylcarbodiimide (500 mg, 2.4 mmol, 1.2 eq.) were added to a solution of 2-acetamidoacrylic acid **12** (260 mg, 2 mmol, 1 eq) and benzylamine (220 µL, 2 mmol, 1 eq) in dry EtOAc (15 mL) at 0 °C. The reaction mixture was then stirred at room temperature for 2 days. The precipitated dicyclohexylurea was then filtered off and the filtrate was evaporated to dryness. The crude was purified by column chromatography (n-heptane/EtOAc: 5/5 to 3/7) to give **13** as a white solid (260 mg, 59 %). **^1^H (CDCl_3_, 400 MHz):** 8.07 (br s, 1H, N*H*Ac), 7.30-7.19 (m, 5H, 5 *H*-Ar), 6.62 (t, 1H, *J* = 5.7 Hz, N*H*Bn), 6.36 (s, 1H, C=C*H*_2_), 5.16 (s, 1H, C=C*H*_2_), 4.44 (d, 2H, *J* = 5.7 Hz, C*H*_2_-Ph), 2.02 (s, 3H, C*H*_3_). **^13^C (CDCl_3_, 100 MHz):** 169.4 (*C*=O), 164.1 (*C*=O), 137.6 (*C*_quat_-Ar or *C*=CH_2_), or 134.4 (*C*_quat_-Ar or *C*=CH_2_), 129.0 (2 *C*-Ar), 128.0 (*C*-Ar), 127.9 (2 *C*-Ar), 101.2 (C=*C*H_2_), 44.3 (*C*H_2_Ph), 24.9 (*C*H_3_). **HRMS (ESI):** m/z calcd for C_12_H_15_N_2_O_2_ [M+H]^+^: 219.1130; found: 219.1128.

**L-γ-glutamyl-2,3-didehydroalanylglycine 14**

1,4-Diiodobutane (28 µL, 0.2 mmol, 1 eq) was added to a solution of glutathione (62 mg, 0.2 mmol, 1 eq) and K_2_CO_3_ (56 mg, 0.4 mmol, 2 eq) in DMF (2 mL) and H_2_O (1 mL) and the resulting solution was stirred at room temperature for 10 minutes and 40 °C for 1 hour. The solvents were then removed under reduced pressure and the crude residue was washed 3 times with acetonitrile to give **14** as a white solid (54 mg, quantitative). **^1^H (D_2_O, 400 MHz):** 5.60 (d, 1H, *J* = 1.0 Hz, *H*-C=C), 5.54 (d, 1H, *J* = 1.0 Hz, *H*-C=C), 3.67 (s, 2H, C*H*_2_), 3.35 (m, 1H, *H-*α), 2.36-2.31 (m, 2H, C*H*_2_), 1.92-1.81 (m, 2H, C*H*_2_). **^13^C (D_2_O, 100 MHz):** 178.6 (*C*=O), 176.4 (*C*=O), 174.8 (*C*=O), 166.4 (*C*=O), 135.3 (C=*C*H_2_), 112.6 (*C*=CH_2_), 54.8 (*C-*α), 43.4 (*C*H_2_), 32.0 (*C*H_2_), 28.3 (*C*H_2_). **LC-MS (ESI):** *m/z*: 274.1 [M+H]^+^

**c(RGDfDha) 15**

1,4-Diiodobutane (0.60 µL, 4.3 µmol, 1 eq) was added to a solution of c(RGDfC) (2.5 mg, 4.3 µmol, 1 eq) and K_2_CO_3_ (1.8 mg, 13.0 µmol, 3 eq) in DMF (1 mL) and H_2_O (0.5 mL) and the resulting solution was stirred at room temperature for 10 minutes and at 40 °C for 1 hour. The solvents were then removed under reduced pressure and the crude residue was purified by flash chromatography (C-18, H_2_O/MeCN: 95/5 to 70/30) and lyophilized to give **15** as a white solid (1.7 mg, 74 %). **^1^H (D_2_O, 400 MHz):** 7.35-7.25 (m, 3H, 3 *H*-Ar), 7.22-7.19 (m, 2H, 2 *H*-Ar), 5.53 (d, 1H, *J* = 1.0 Hz, *H*_DHA_), 5.01 (d, 1H, *J* = 1.0 Hz, *H*_DHA_), 4.61 (m, 1H, *H*-α), 4.49 (dd, 1H, *J* = 9.0, *J* = 6.8 Hz, *H*-α), 4.41 (dd, 1H, *J* = 8.5, *J* = 6.1 Hz, *H*-α), 4.15 (d, 1H, *J* = 14.7 Hz, C*H*H-Ph), 3.46 (d, 1H, *J* = 14.5 Hz, C*H*H-Ph), 3.13 (td, 2H, *J* = 6.9, *J* = 1.9 Hz, C*H*_2_), 3.08 (dd, 1H, *J* = 13.4, *J* = 6.2 Hz, C*H*H), 2.96 (dd, 1H, *J* = 13.4, *J* = 8.9 Hz, C*H*H), 2.56 (dd, 1H, *J* = 15.8, *J* = 7.2 Hz, C*H*H), 2.44 (dd, 1H, *J* = 15.8, *J* = 7.6 Hz, C*H*H), 1.84 (m, 1H, C*H*H), 1.67-1.45 (m, 3H, C*H*_2_, C*H*H). **HRMS (ESI):** m/z calcd for C_24_H_33_N_8_O_7_ [M+H]^+^: 545.2467; found: 545.2465.

**2-Acetamido-*N*-benzyl-3-((2-(2-(2-((2-fluoropyridin-3-yl)oxy)ethoxy)ethoxy)ethyl)thio)propanamide 16**

**4** (65 mg, 0.25 mmol, 1 eq) was added to a solution of **13** (55 mg, 0.25 mmol, 1 eq) in acetonitrile (3 mL) and 0.1 M NaHCO_3_ pH 8.3 (3 mL) and the resulting solution was stirred at room temperature overnight. After completion, EtOAc (10 mL) was added and the organic layer was washed with H_2_O (5 mL) and brine (5 mL), dried over anhydrous Na_2_SO_4_, and concentrated under reduced pressure. The residue was purified by flash chromatography (n-heptane/EtOAc: 5/5 to 100 % EtOAc) to give **16** as a colorless oil (100 mg, 83 %). **^1^H (CDCl_3_, 400 MHz):** 7.74 (td, 1H, *J*_5,6_ = 4.9, *J*_4,6_ = *J*_H-F_ = 1.7 Hz, *H*-6), 7.33-7.2 (m, 7H, 5 *H*-Ar, Bn-N*H*, *H*-4), 7.09 (ddd, 1H, *J*_4,5_ = 7.9, *J*_5,6_ = 4.9, *J*_H-F_ = 0.7 Hz, *H*-5), 6.72 (d, 1H, *J* = 7.6 Hz, N*H*Ac), 4.63 (td, 1H, *J* = 7.6, *J* = 5.8 Hz, *H-*α), 4.46 (dd, 1H, *J*_gem_ = 14.8, *J* = 6.0 Hz, C*H*_2_-Ph), 4.40 (dd, 1H, *J*_gem_ = 14.8, *J* = 6.0 Hz, C*H*_2_-Ph), 4.11 (t, 2H, *J* = 4.8 Hz, C*H*_2_), 3.84-3.75 (m, 2H, C*H*_2_), 3.73-3.61 (m, 4H, 2 C*H*_2_), 3.60-3.52 (m, 2H, C*H*_2_), 2.99 (dd, 1H, *J* = 14.0, *J* = 5.8 Hz, Cα-C*H*H-S), 2.83 (dd, 1H, *J* = 14.0, *J* = 7.6 Hz, Cα-C*H*H-S), 2.80-2.77 (m, 2H, C*H*_2_-S), 2.00 (s, 3H, C*H*_3_). **^13^C (CDCl_3_, 100 MHz):** 170.4 (*C*=O), 170.1 (*C*=O), 153.8 (d, ^1^*J*_C-F_ = 238.9 Hz, *C*_2_-Ar), 142.1 (d, ^2^*J*_C-F_ = 25.5 Hz, *C*_3_-Ar), 137.9 (*C*_quat_-Ar), 137.7 (d, ^3^*J*_C-F_ = 13.1 Hz, *C*_6_-Ar), 128.6 (2 *C*-Ar), 127.7 (2 *C*-Ar), 127.5 (*C*-Ar), 123.4 (d, ^3^*J*_C-F_ = 4.1 Hz, *C*_4_-Ar), 121.8 (d, ^4^*J*_C-F_ = 4.2 Hz, *C*_5_-Ar), 71.2 (*C*H_2_), 70.8 (*C*H_2_), 70.1 (*C*H_2_), 69.3 (*C*H_2_), 69.0 (*C*H_2_), 53.1 (*C-*α), 43.6 (*C*H_2_Ph), 34.9 (*C*H_2_-S), 32.5 (*C*H_2_-S), 23.2 (*C*H_3_). **HRMS (ESI):** m/z calcd for C_23_H_31_FN_3_O_5_S [M+H]^+^: 480.1963; found: 480.1963.

**L-γ-glutamyl-(2-(2-(2-((2-fluoropyridin-3-yl)oxy)ethoxy)ethoxy)thioethyl)cysteinylglycine 17**

**4** (26 mg, 0.1 mmol, 1 eq) was added to a solution of **14** (27 mg, 0.1 mmol, 1 eq) in DMF (0.5 mL) and 0.1 M NaHCO_3_ pH 8.3 (0.5 mL) and the resulting solution was stirred at room temperature overnight. After completion of the reaction, the solvents were removed under reduced pressure and the crude residue was purified by flash chromatography (C-18, H_2_O/MeCN: 95/5 to 75/25) and lyophilized to give **17** as an off-white solid (41 mg, 77 %). **^1^H (D_2_O, 400 MHz):** 7.76 (td, 1H, *J*_5,6_ = 5.0, *J*_4,6_ = *J*_H-F_ = 1.5 Hz, *H*-6), 7.66 (ddd, 1H, *J*_4,F_ = 10.1, *J*_4,5_ = 8.4, *J*_4,6_ = 1.5 Hz, *H*-4), 7.33 (ddd, 1H, *J*_4,5_ = 8.4, *J*_5,6_ = 5.0, *J*_5,F_ = 0.4 Hz, *H*-5), 4.59 (ddd, 1H, *J* = 9.0, *J* = 4.9, *J* = 2.1 Hz, *H*-α), 4.36-4.34 (m, 2H, C*H*_2_), 3.97-3.95 (m, 2H, C*H*_2_), 3.82-3.63 (m, 9H, 4 C*H*_2_, *H*-α), 3.11 (ddd, 1H, *J* = 14.0, *J* = 5.1, *J* = 2.5 Hz, C*H*H), 2.89 (dd, 1H, *J* = 14.0, *J* = 8.9 Hz, C*H*H), 2.80 (td, 2H, *J* = 6.2, *J* = 2.5 Hz, C*H*_2_), 2.53-2.48 (m, 2H, C*H*_2_), 2.16-2.03 (m, 2H, C*H*_2_). **^13^C (D_2_O, 100 MHz):** 176.1 (2 *C*=O), 175.7 (*C*=O), 171.9 (*C*=O), 153.5 (d, ^1^*J*_C-F_ = 238.6 Hz, *C*_2_-Ar), 141.5 (d, ^2^*J*_C-F_ = 23.9 Hz, *C*_3_-Ar), 137.0 (d, ^3^*J*_C-F_ = 11.7 Hz, *C*_6_-Ar), 125.0 (d, ^3^*J*_C-F_ = 3.9 Hz, *C*_4_-Ar), 122.7 (d, ^4^*J*_C-F_ = 3.8 Hz, *C*_5_-Ar), 69.8 (*C*H_2_), 69.6 (*C*H_2_), 69.4 (*C*H_2_), 68.7 (*C*H_2_), 68.5 (*C*H_2_), 54.8 (*C-*α), 53.1 (*C-*α), 43.3 (*C*H_2_), 33.1 (*C*H_2_), 31.9 (*C*H_2_), 31.0 (*C*H_2_), 28.6 (*C*H_2_). **HRMS (ESI):** m/z calcd for C_21_H_32_FN_4_O_9_S [M+H]^+^: 535.1869; found: 535.1869.

**c(RGDf-*S*-2-(2-(2-((2-fluoropyridin-3-yl)oxy)ethoxy)ethoxy)ethyl)cysteine) 18**

**4** (1.0 mg, 3.7 µmol, 1 eq) was added to a solution of **15** (2.0 mg, 3.7 µmol, 1 eq) in DMF (100 µL) and 0.1 M NaHCO_3_ pH 8.3 (300 µL) and the resulting solution was stirred at room temperature for 5 hours. After completion of the reaction, the solvents were removed under reduced pressure and the crude residue was purified by flash chromatography (C-18, H_2_O/MeCN: 95/5 to 40/60) and lyophilized to give **18** as a white solid (2.4 mg, 80 %). **^1^H (D_2_O, 400 MHz):** 7.73 (td, 1H, *J*_5,6_ = 4.8, *J*_4,6_ = *J*_H-F_ = 1.4 Hz, *H*-6), 7.62 (ddd, 1H, *J*_4,F_ = 10.4, *J*_4,5_ = 8.4, *J*_4,6_ = 1.4 Hz, *H*-4), 7.40-7.24 (m, 6H, 5 *H*-Ar, *H*-5), 4.44 (dd, 1H, *J* = 9.2, *J* = 3.7 Hz, *H*-α), 4.40 (dd, 1H, *J* = 8.4, *J* = 6.8 Hz, *H*-α), 4.32 (dd, 2H, *J* = 5.5, *J* = 3.0 Hz, C*H*_2_), 4.06 (d, 1H, *J* = 15.1 Hz, C*H*H-Ph), 3.94 (dd, 2H, *J* = 6.1, *J* = 2.4 Hz, C*H*_2_), 3.79-3.77 (m, 2H, C*H*_2_), 3.73-3.69 (m, 3H, C*H*_2_, *H*-α), 3.66 (d, 1H, *J* = 15.1 Hz, C*H*H-Ph), 3.21-3.16 (m, 2H, C*H*_2_), 3.11 (d, 2H, *J* = 7.6 Hz, C*H*_2_), 2.89 (dd, 1H, *J* = 13.7, *J* = 4.4 Hz, C*H*H), 2.79-2.75 (m, 2H, C*H*_2_), 2.65 (dd, 1H, *J* = 13.7, *J* = 5.5 Hz, C*H*H), 2.47 (d, 2H, *J* = 7.6 Hz, C*H*_2_), 1.90-1.51 (m, 6H, 3 C*H*_2_). (there is a *H*-α under the residual solvent peak) **HRMS (ESI):** m/z calcd for C_35_H_49_FN_9_O_10_S [M+H]^+^: 806.3302; found: 806.3297.

**c(RGDfK)-PEG_2_-maleimide 20**

**Maleimide-PEG-NHS** **19** (3.8 mg, 9.0 µmol, 1.5 eq) in 100 µL MeCN was added to a solution of **cyclic RGDfK** (5.0 mg, 6.0 µmol, 1 eq) in 0.1 M NaPi pH 7.0 (500 µL) and the resulting solution was stirred at room temperature for 30 minutes. After completion of the reaction, the crude was lyophilized and the solid was washed with MeCN (3 x 500 µL) to give **20** as a white solid (4.8 mg, 87 %). **^1^H (D_2_O, 400 MHz):** 7.30-7.15 (m, 5H, *H*-Ar), 6.76 (s, 2H, *H*C=C*H*), 4.61 (t, 1H, *J* = 7.3 Hz, *H*-α), 4.48 (dd, 1H, *J* = 10.8, *J* = 5.5 Hz, *H*-α), 4.32 (dd, 1H, *J* = 8.7, *J* = 5.8 Hz, *H*-α), 4.13 (d, 1H, *J* = 14.6 Hz, C*H*H-Ph), 3.75 (dd, 1H, *J* = 10.7, *J* = 4.2 Hz, *H*-α), 3.71-3.68 (m, 4H, 2 C*H*_2_), 3.58-3.54 (m, 4H, 2 C*H*_2_), 3.46 (t, 2H, *J* = 5.4 Hz, C*H*_2_), 3.38 (d, 1H, *J* = 14.6 Hz, C*H*H-Ph), 3.22 (t, 2H, *J* = 5.3 Hz, C*H*_2_), 3.13-2.96 (m, 4H, 2 C*H*_2_), 2.86 (dd, 1H, *J* = 13.2, *J* = 10.7 Hz, C*H*H), 2.58 (dd, 1H, *J* = 15.7, *J* = 7.2 Hz, C*H*H), 2.47-2.37 (m, 6H, 3 C*H*_2_), 1.77 (m, 1H, C*H*H), 1.61-1.51 (m, 2H, C*H*_2_), 1.47-1.32 (m, 3H, C*H*_2_, C*H*H), 1.27-1.19 (m, 2H, C*H*_2_), 0.81-0.71 (m, 2H, C*H*_2_). **HRMS (ESI):** m/z calcd for C_41_H_60_N_11_O_13_ [M+H]^+^: 914.4367; found: 914.4373.

**c(RGDfK)-PEG_2_-maleimide-SFPy 21**

**4** (0.8 mg, 3.0 µmol, 1.5 eq) in 100 µL MeCN was added to a solution of **20** (1.8 mg, 2.0 µmol, 1 eq) in 0.1 M NaPi pH 7.0 (500 µL) and the resulting solution was stirred at room temperature for 1 hour. After completion of the reaction, the crude was lyophilized and the solid was washed with MeCN (3 x 500 µL) to give **21** as a white solid (2.0 mg, 86 %). **^1^H (D_2_O, 400 MHz):** 7.64 (m, 1H, *H*-6), 7.53 (ddd, 1H, *J*_H-F_ = 10.2, *J*_4,5_ = 8.7, *J*_4,6_ = 1.8 Hz, *H*-4), 7.29-7.13 (m, 6H, *H*-Ar, *H*-5), 4.61 (t, 1H, *J* = 7.8 Hz, *H*-α), 4.47 (dd, 1H, *J* = 10.7, *J* = 5.2 Hz, *H*-α), 4.31 (dd, 1H, *J* = 9.2, *J* = 5.6 Hz, *H*-α), 4.23-4.20 (m, 2H, C*H*_2_), 4.12 (d, 1H, *J* = 14.4 Hz, C*H*H-Ph), 3.85-3.82 (m, 2H, C*H*_2_), 3.75-3.60 (m, 11H, 5 C*H*_2_, *H*-α), 3.56 (br s, 4H, 2 C*H*_2_), 3.47 (t, 2H, *J* = 5.2 Hz, C*H*_2_), 3.38 (d, 1H, *J* = 14.4 Hz, C*H*H-Ph), 3.24-2.96 (m, 7H, 3 C*H*_2_, C*H*), 2.80 (m, 1H, C*H*H), 2.60-2.55 (m, 5H, 2 C*H*_2_, C*H*H), 2.46-2.35 (m, 6H, 3 C*H*_2_), 1.76 (m, 1H, C*H*H), 1.60-1.50 (m, 2H, C*H*_2_), 1.45-1.28 (m, 3H, C*H*_2_, C*H*H), 1.24-1.18 (m, 2H, C*H*_2_), 0.78-0.70 (m, 2H, C*H*_2_). **HRMS (ESI):** m/z calcd for C_52_H_76_FN_12_O_16_S [M+H]^+^: 1175.5202; found: 1175.5193.

# II. ^1^H and ^13^C NMR spectra

**2, CDCl_3_**


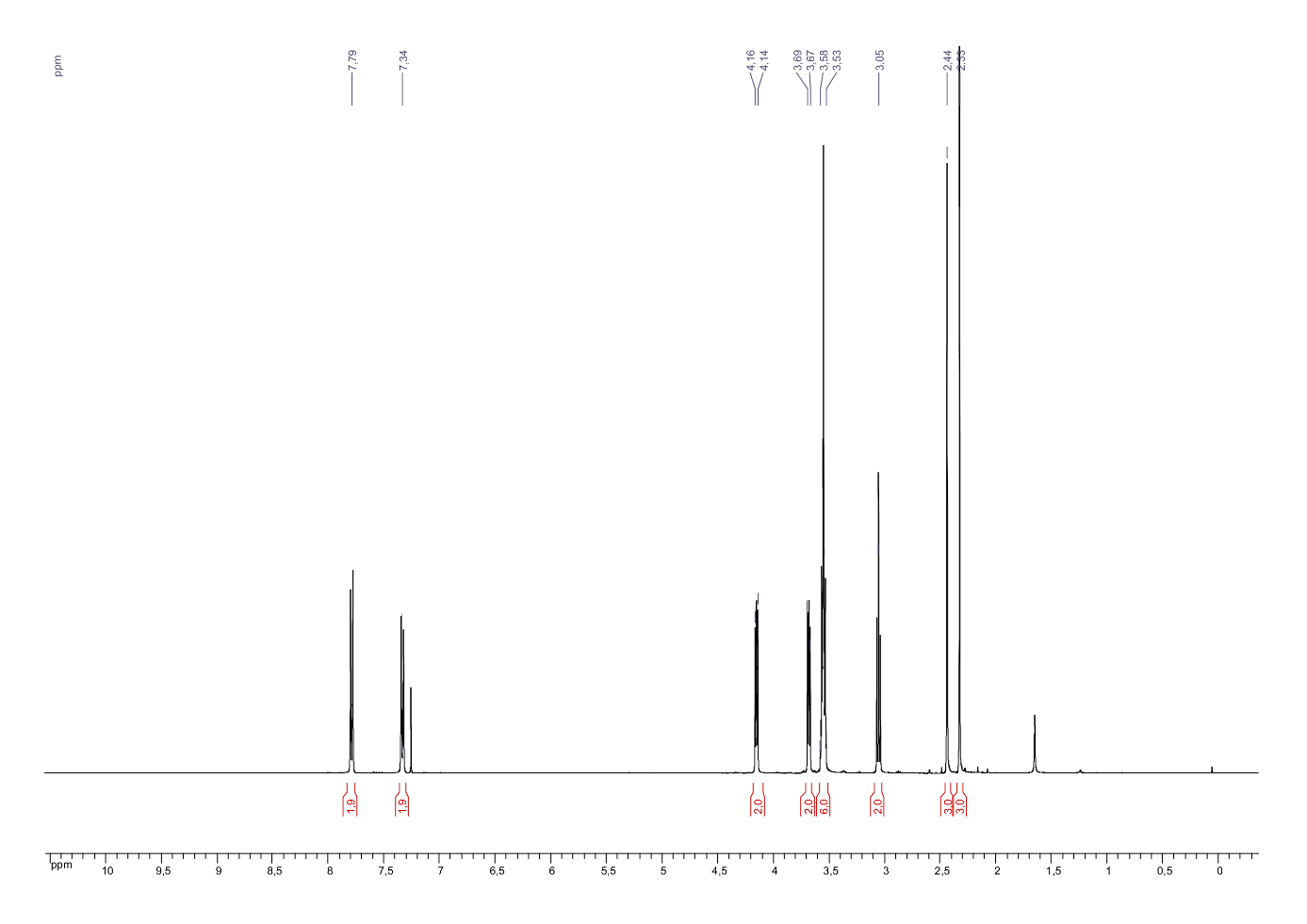

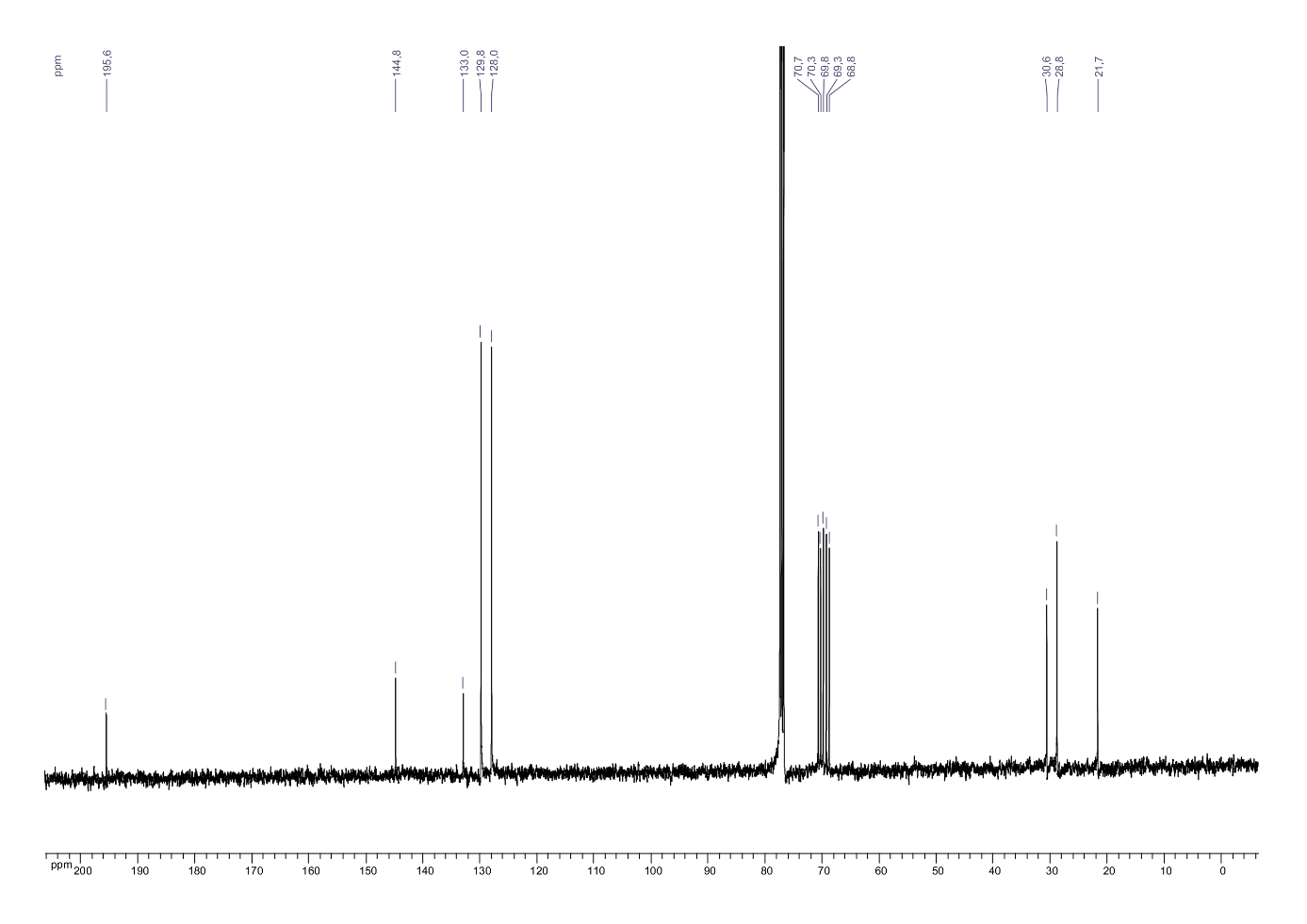


**3, CDCl_3_**


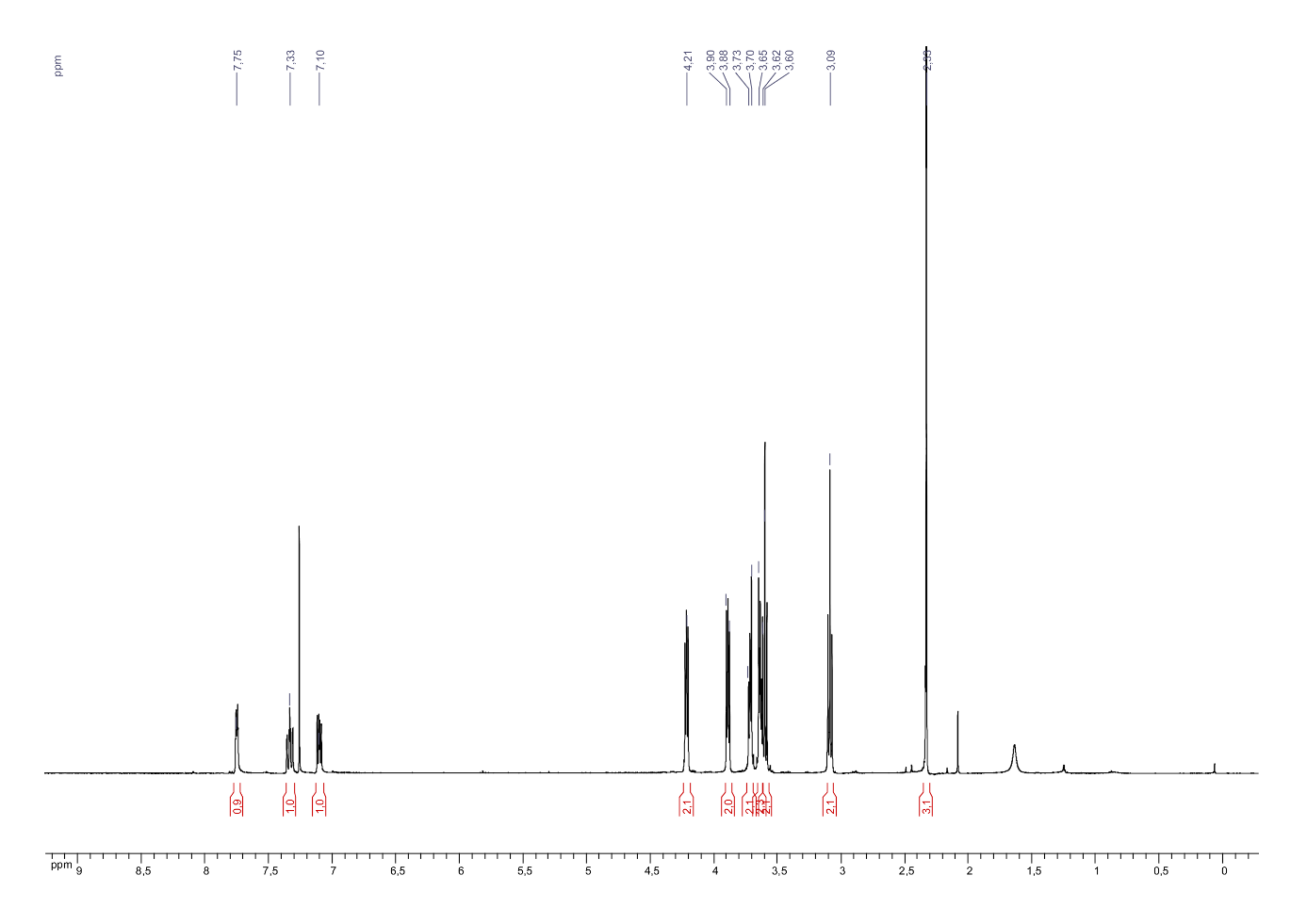

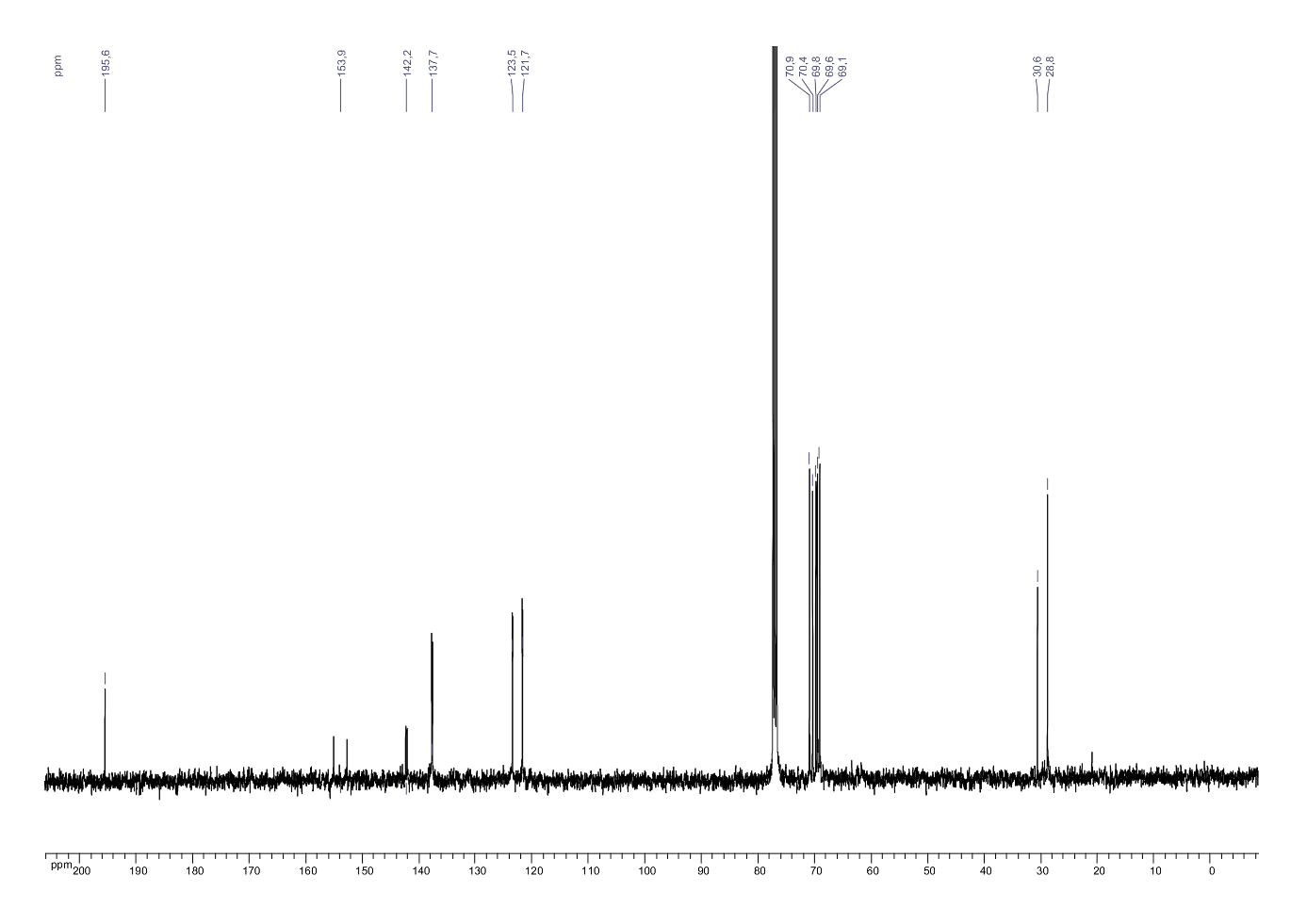


**4, CDCl_3_**

**
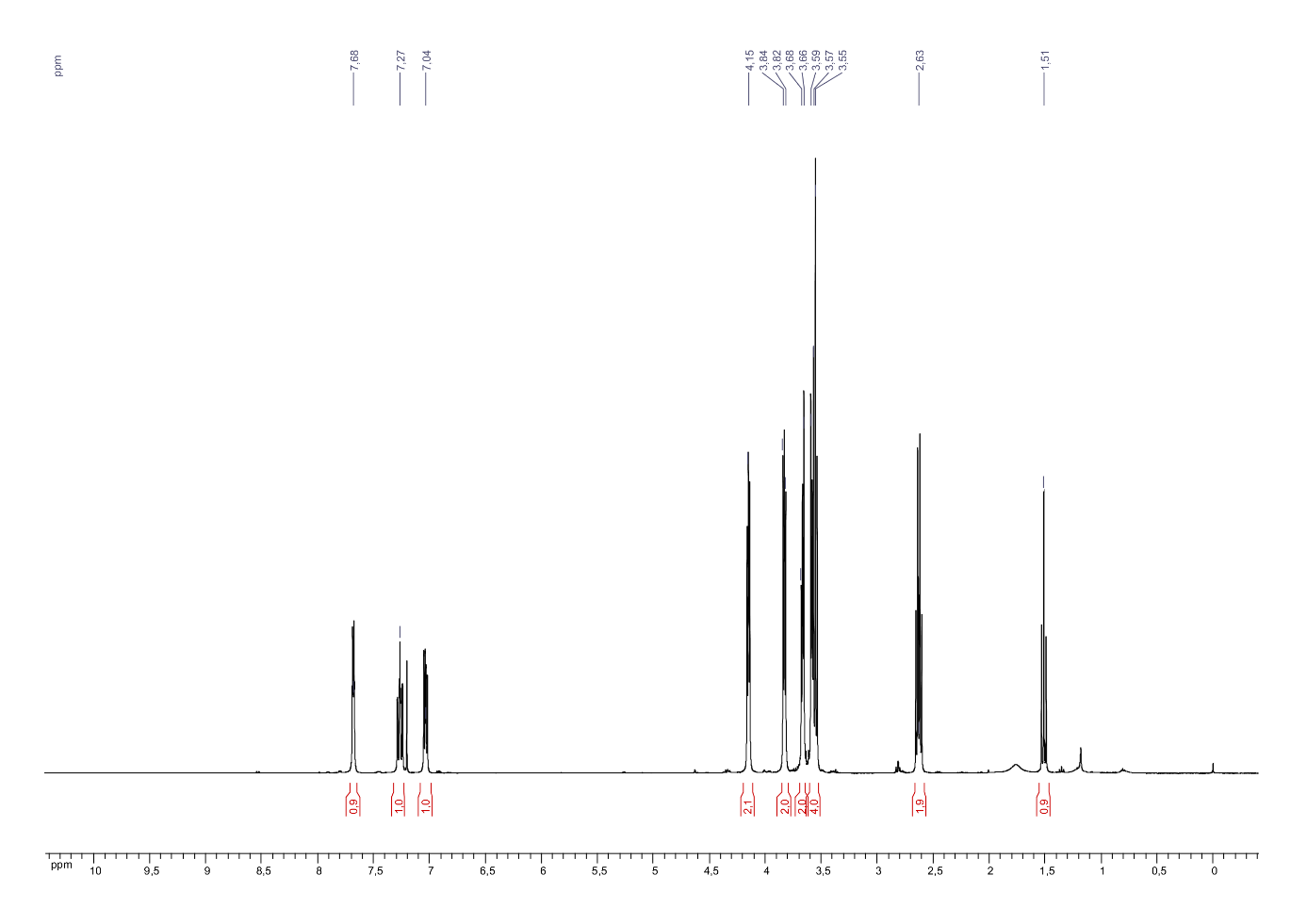
** **
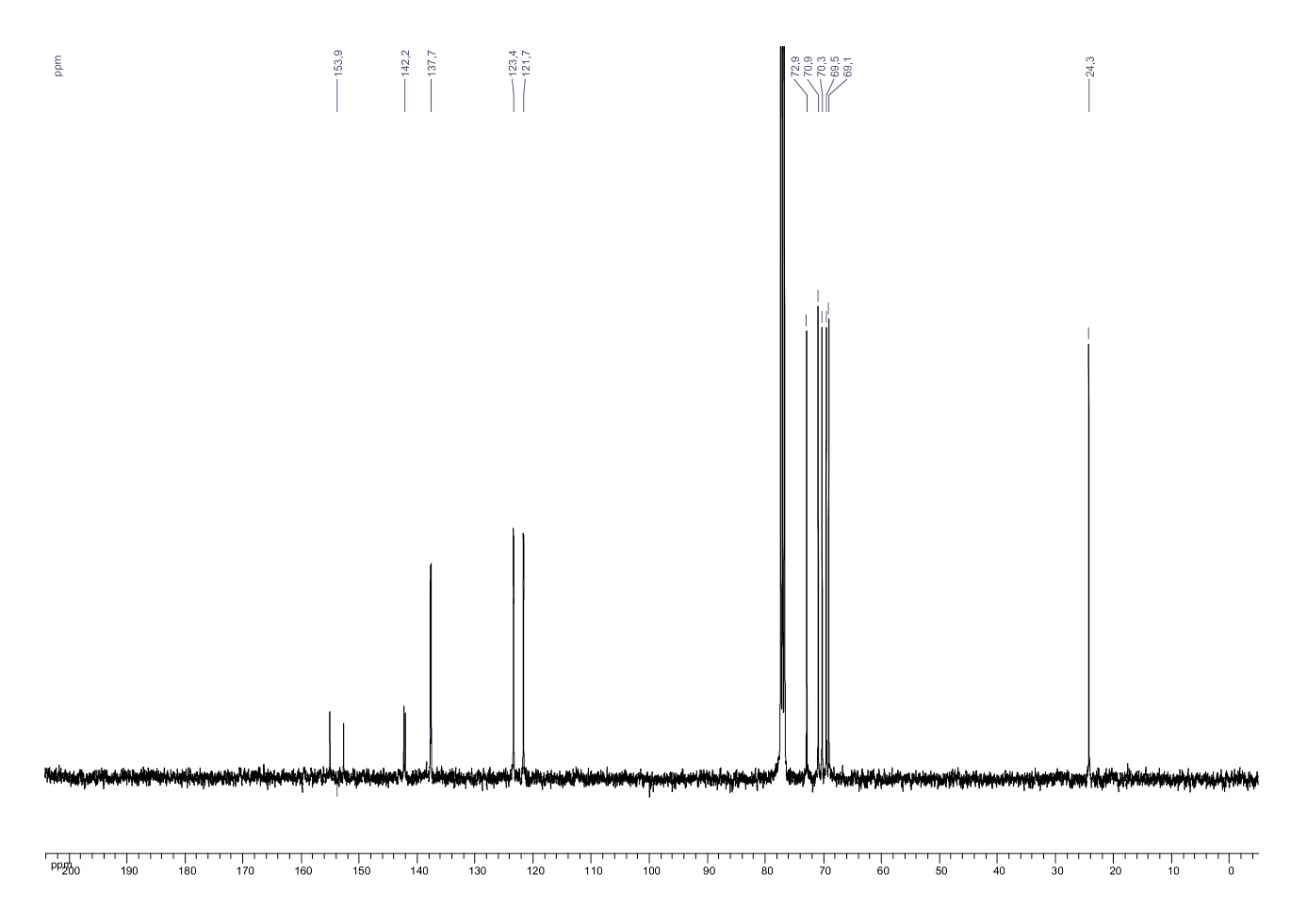
**

**6, CDCl_3_**


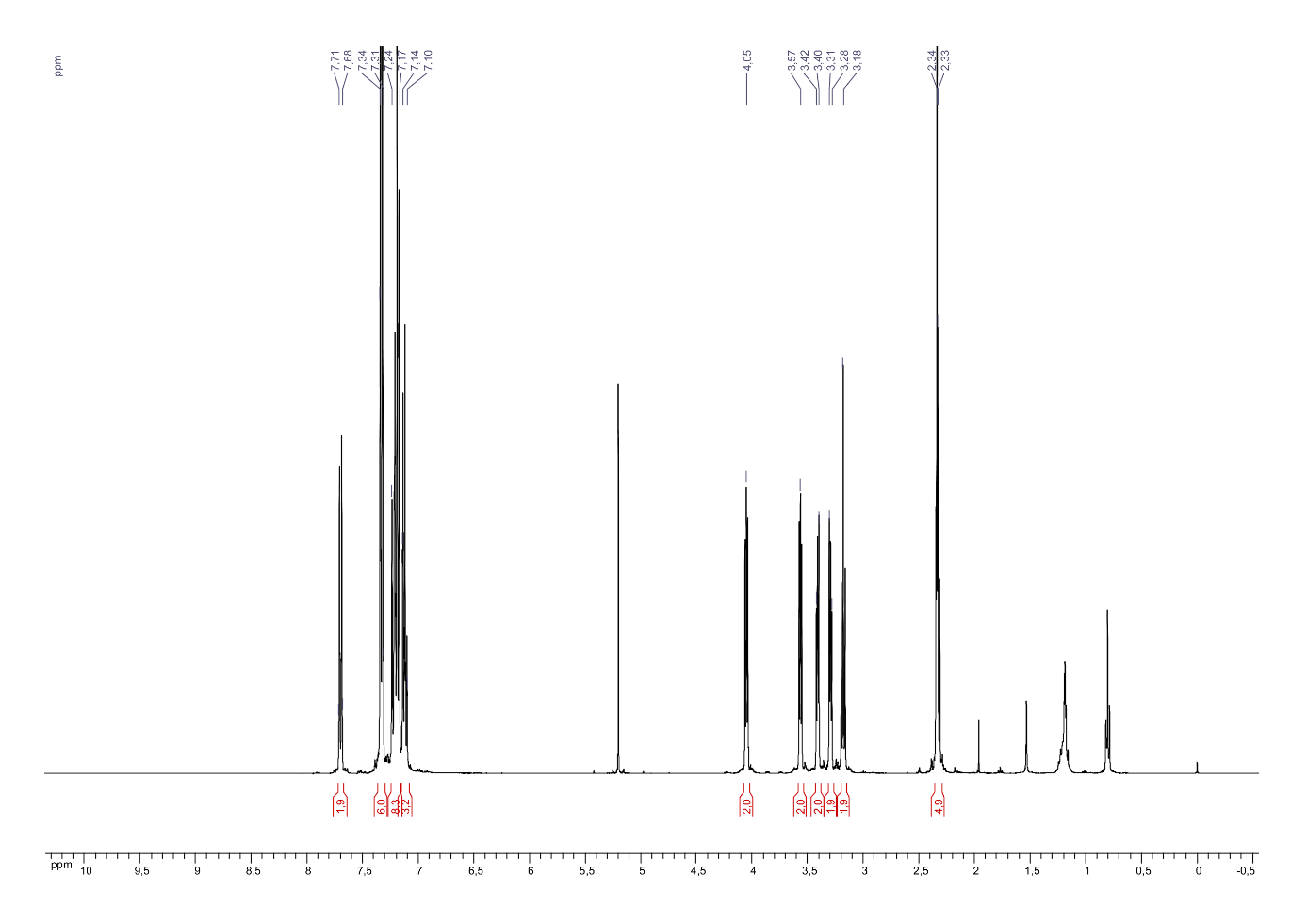

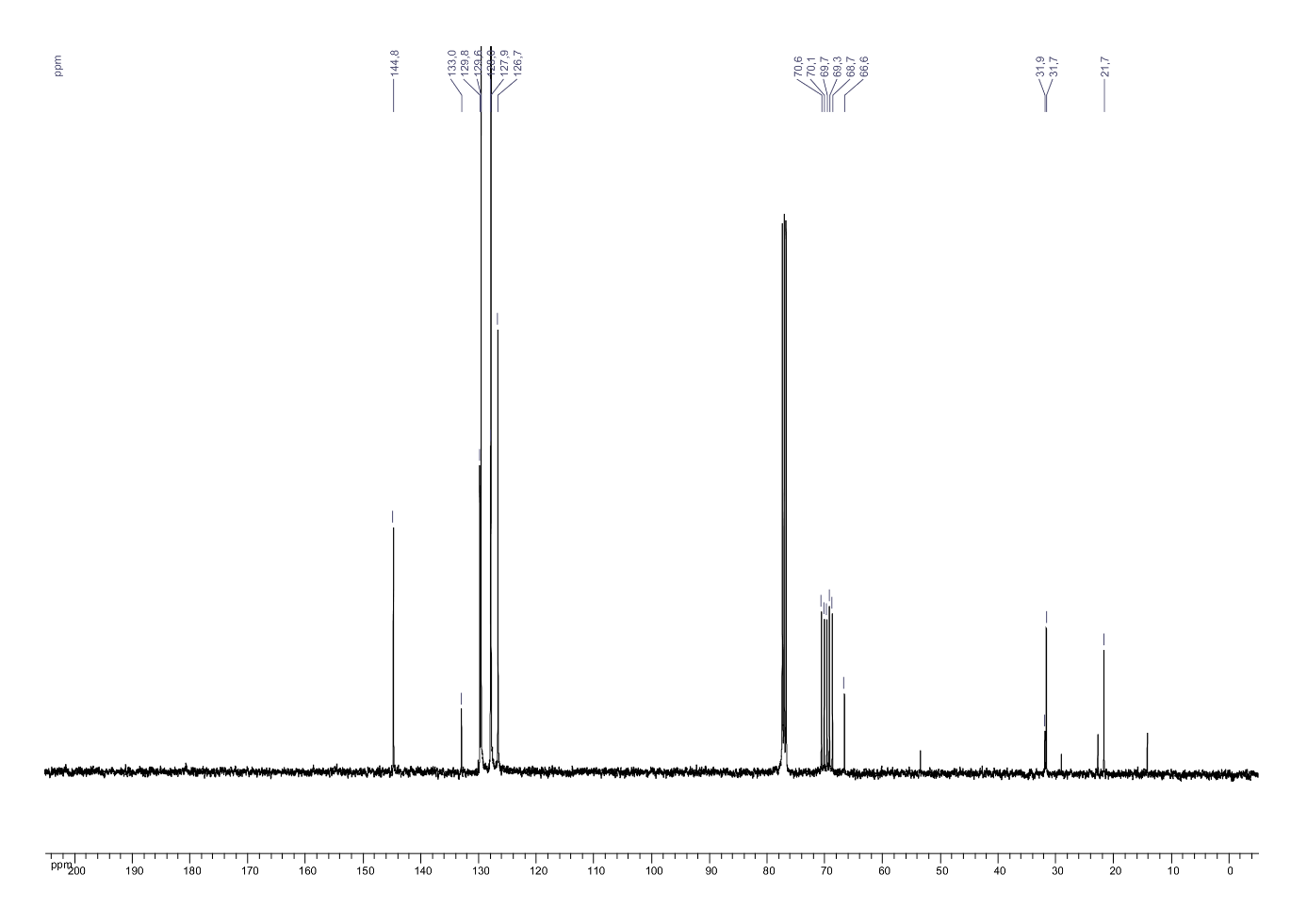


**7, CDCl_3_**

**
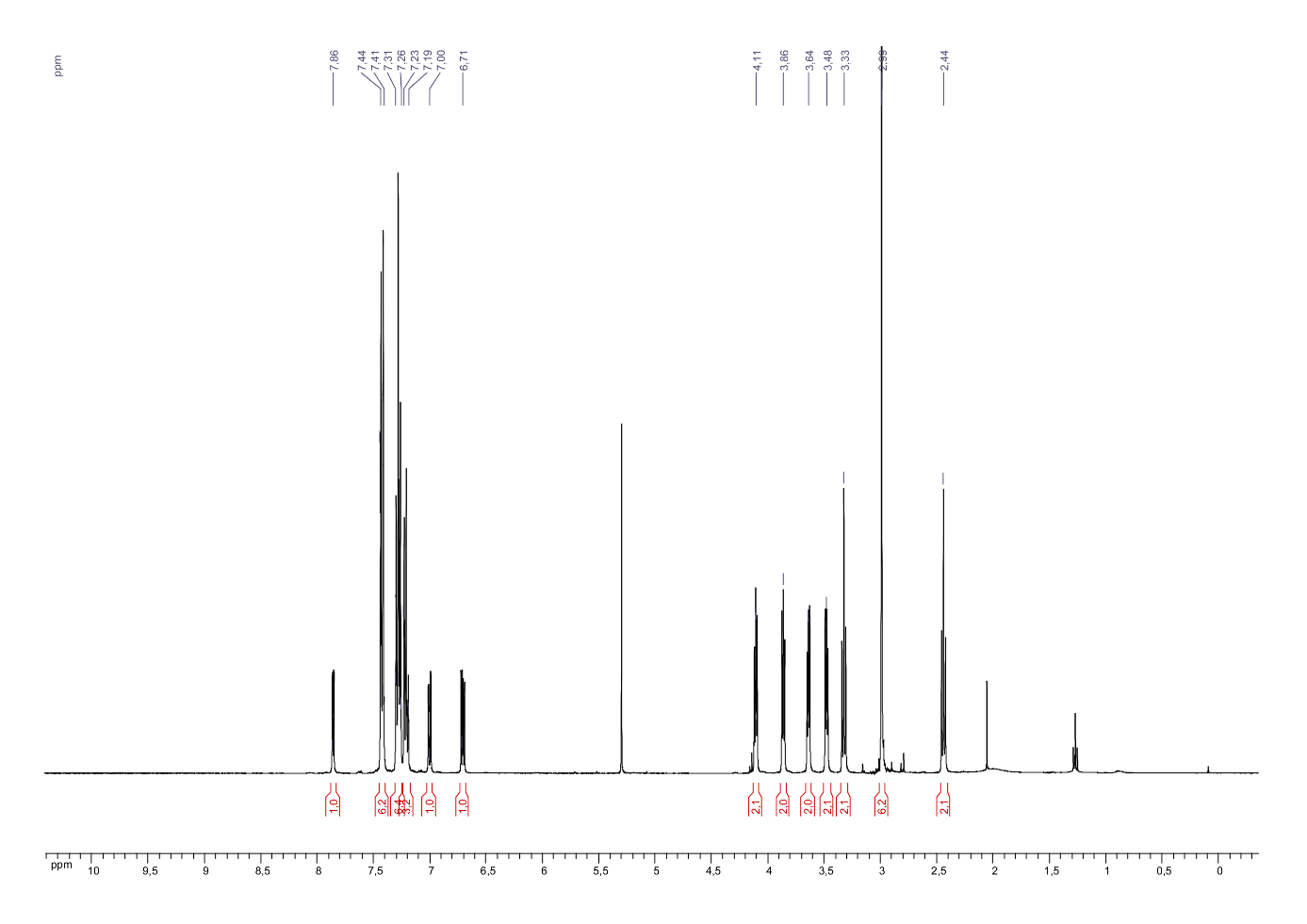
** **
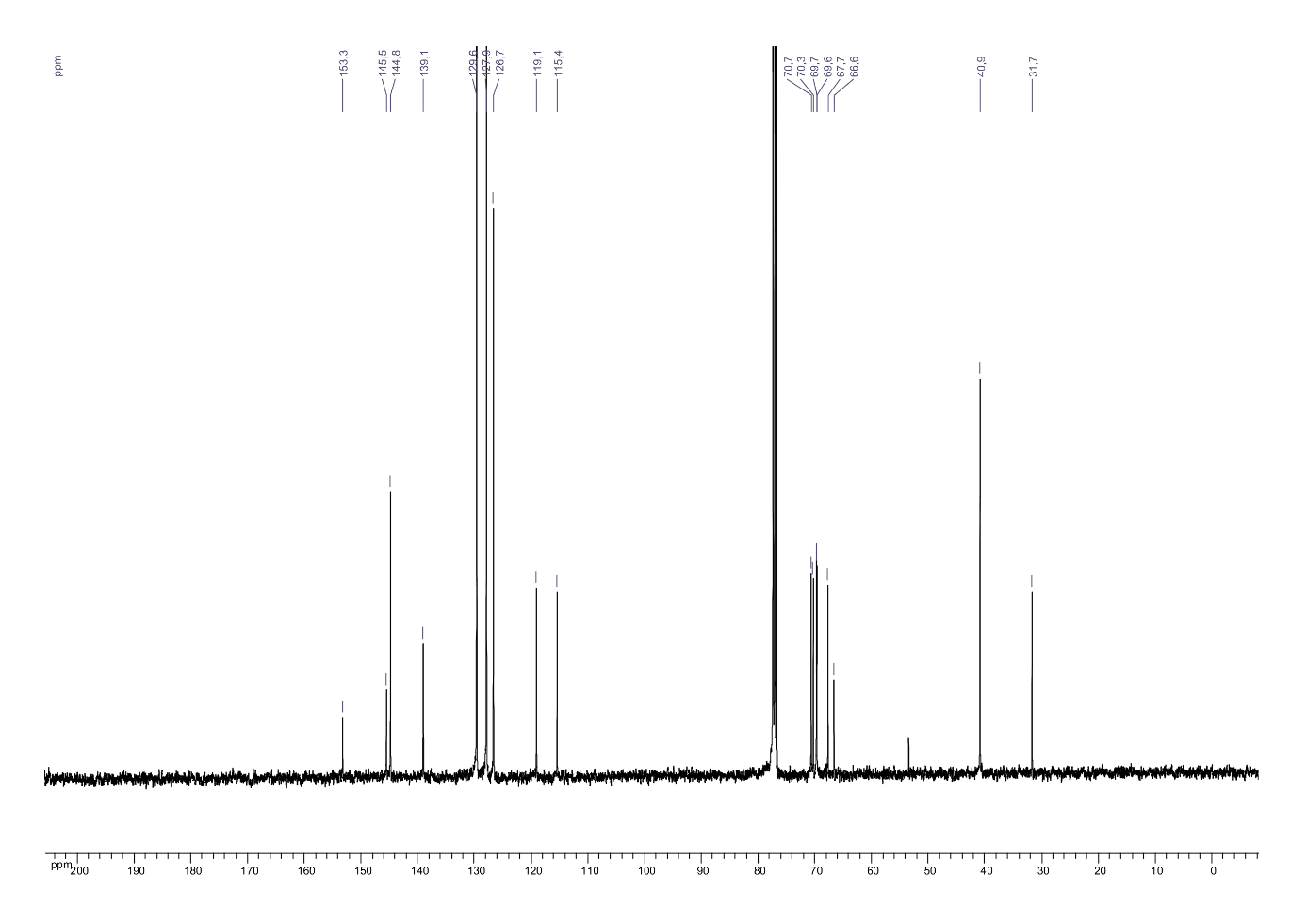
**

**8, CDCl_3_**

**
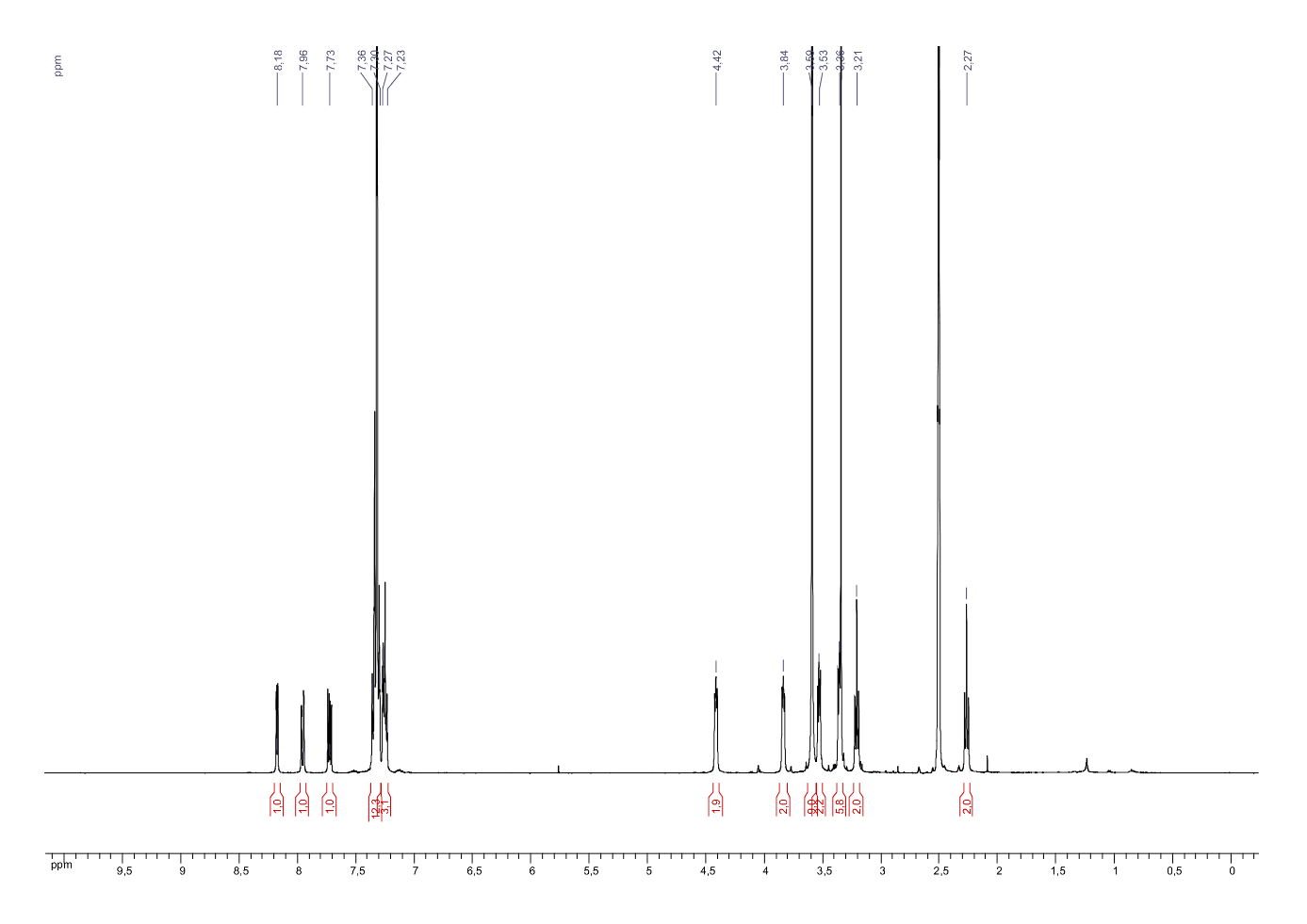
**
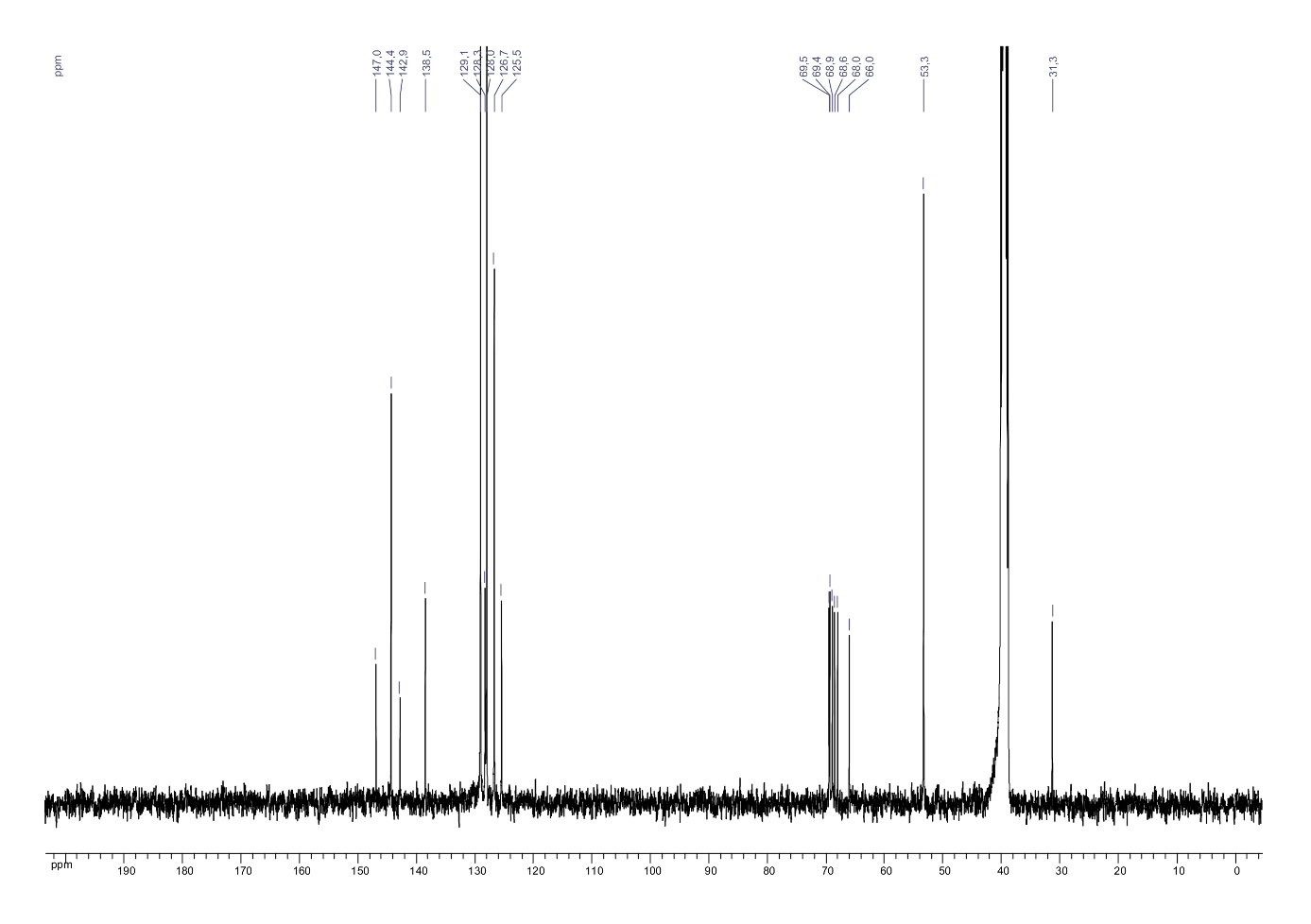


**9, CDCl_3_**

**
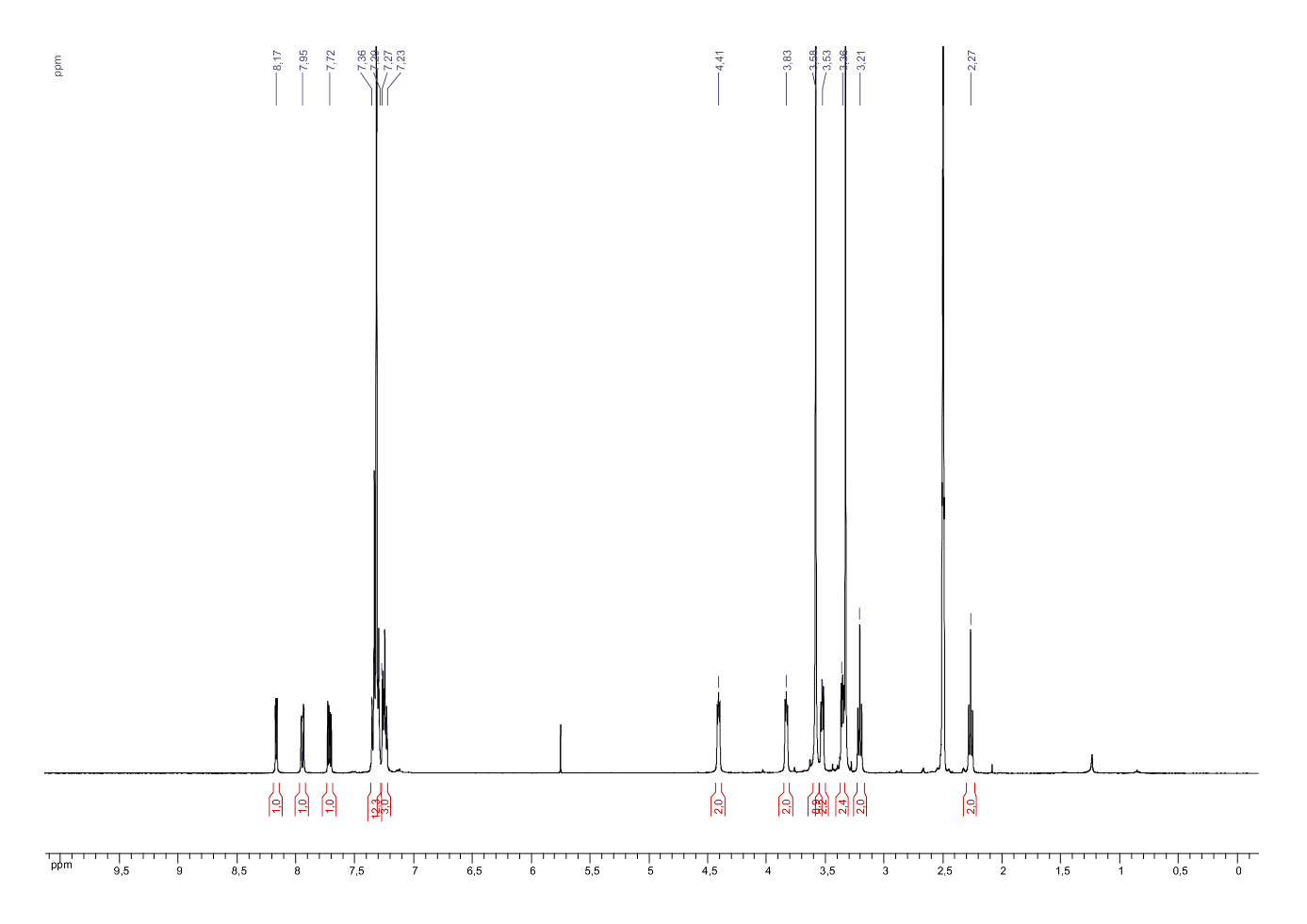
** **
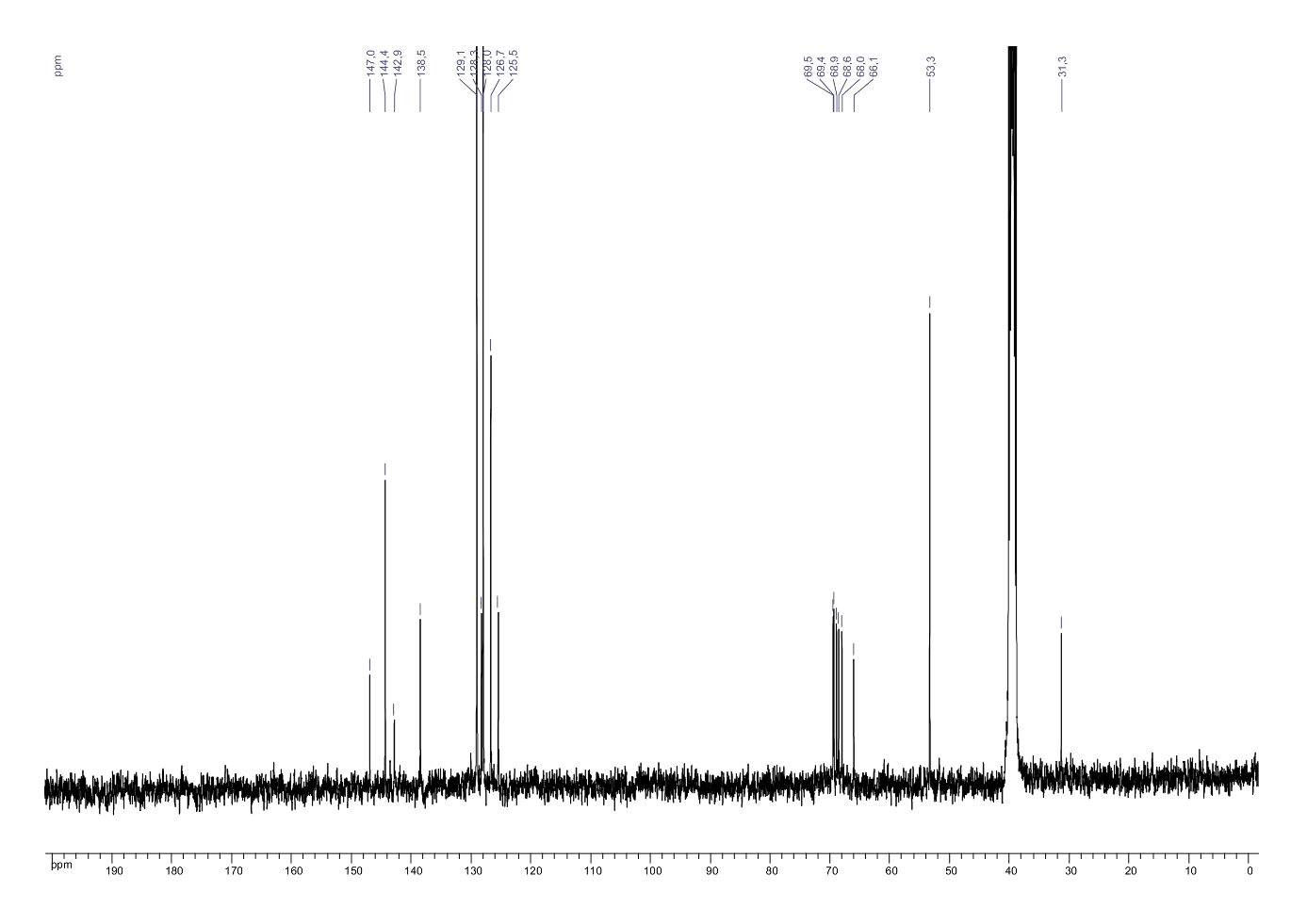
**

**12, CDCl_3_**

**
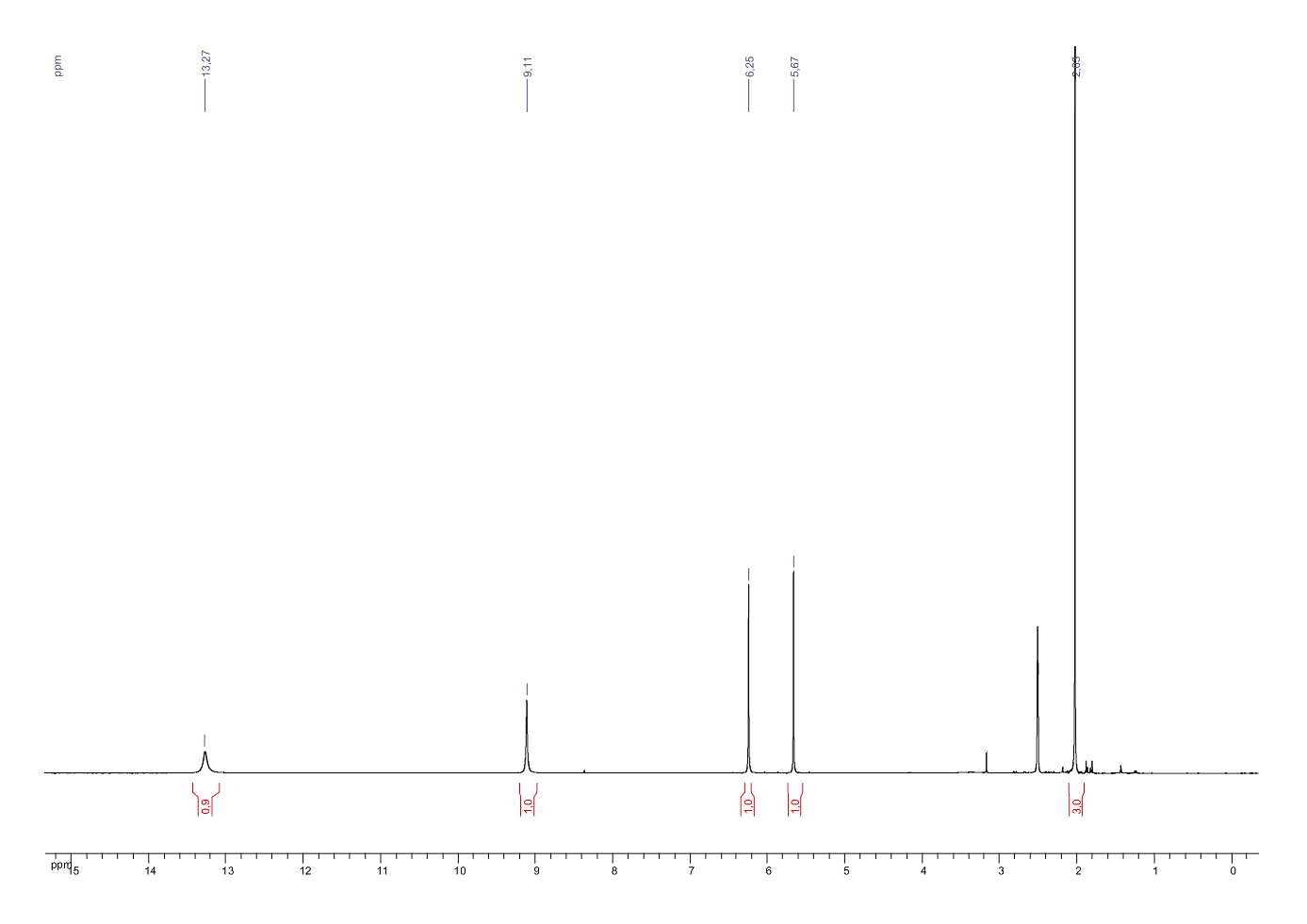
** **
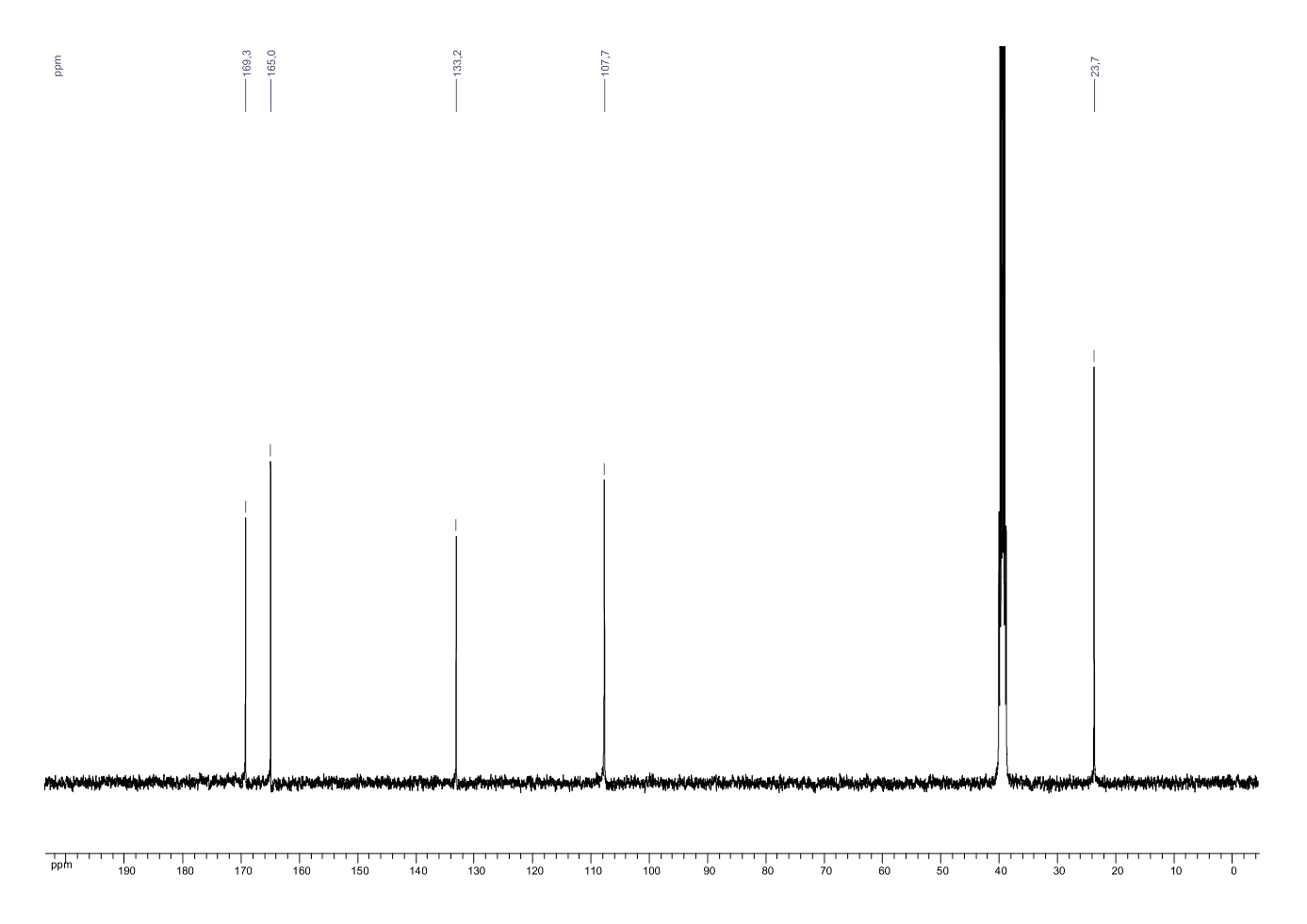
**

**13, CDCl_3_**

**
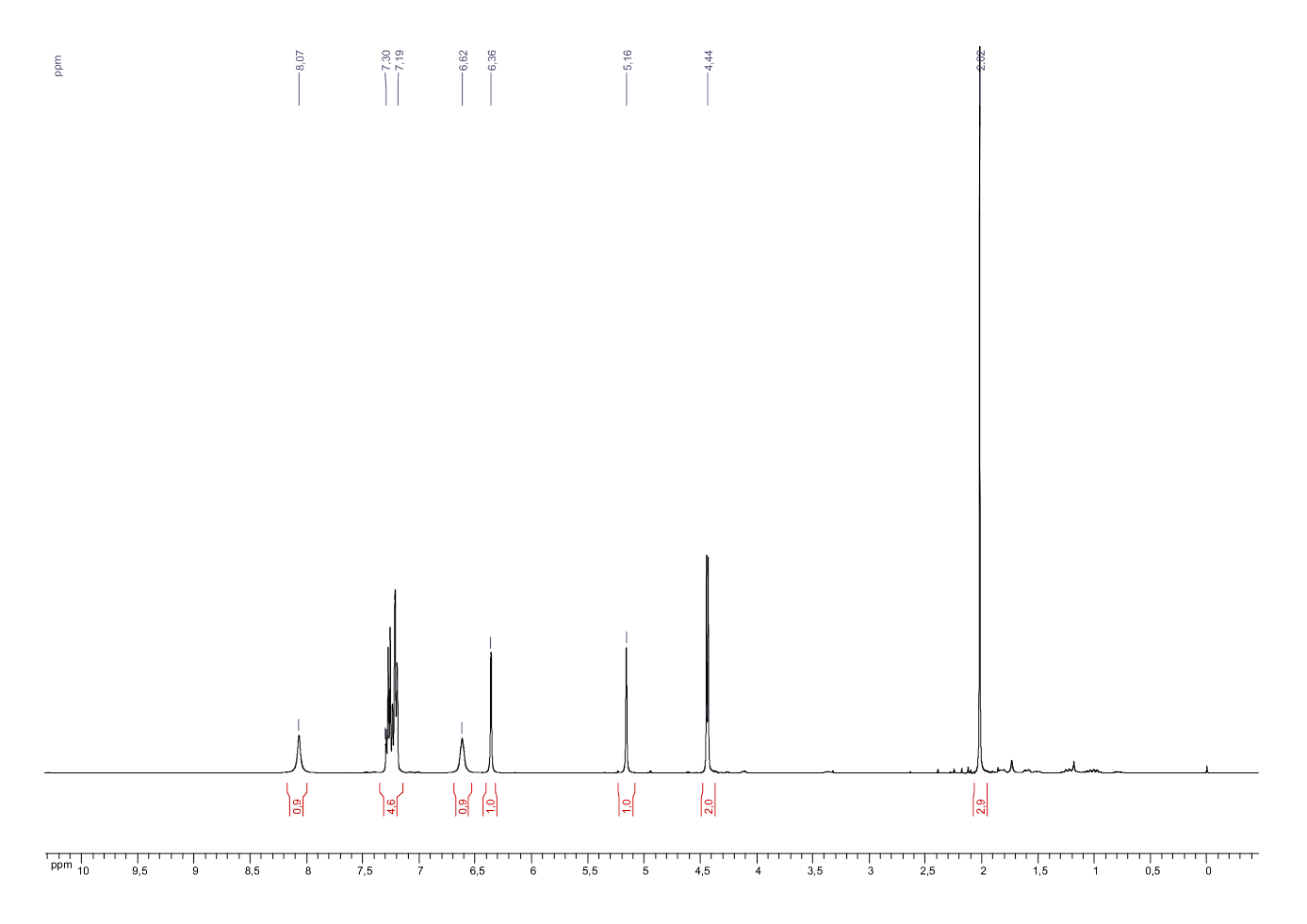
** **
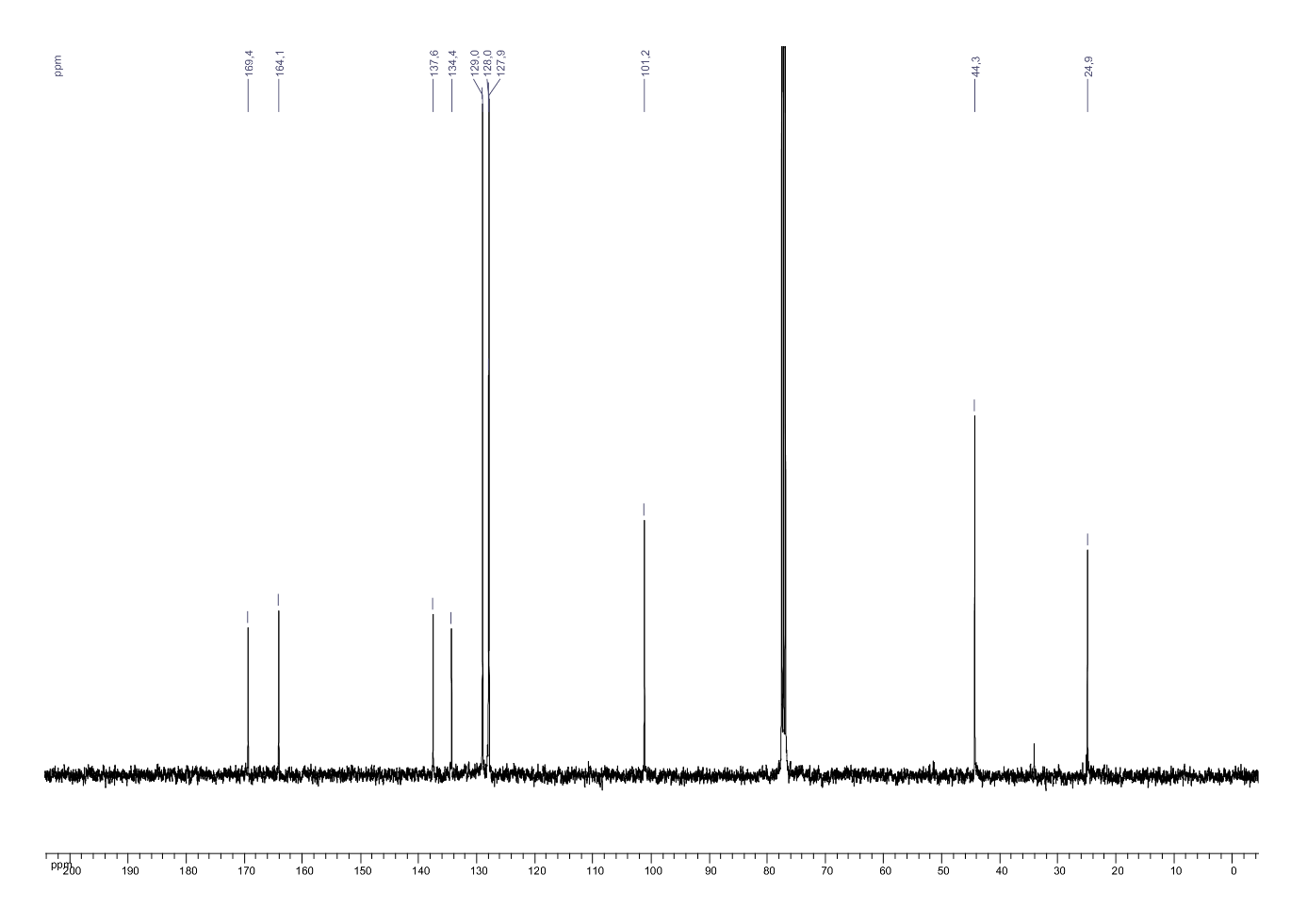
**

**14, D_2_O**

**
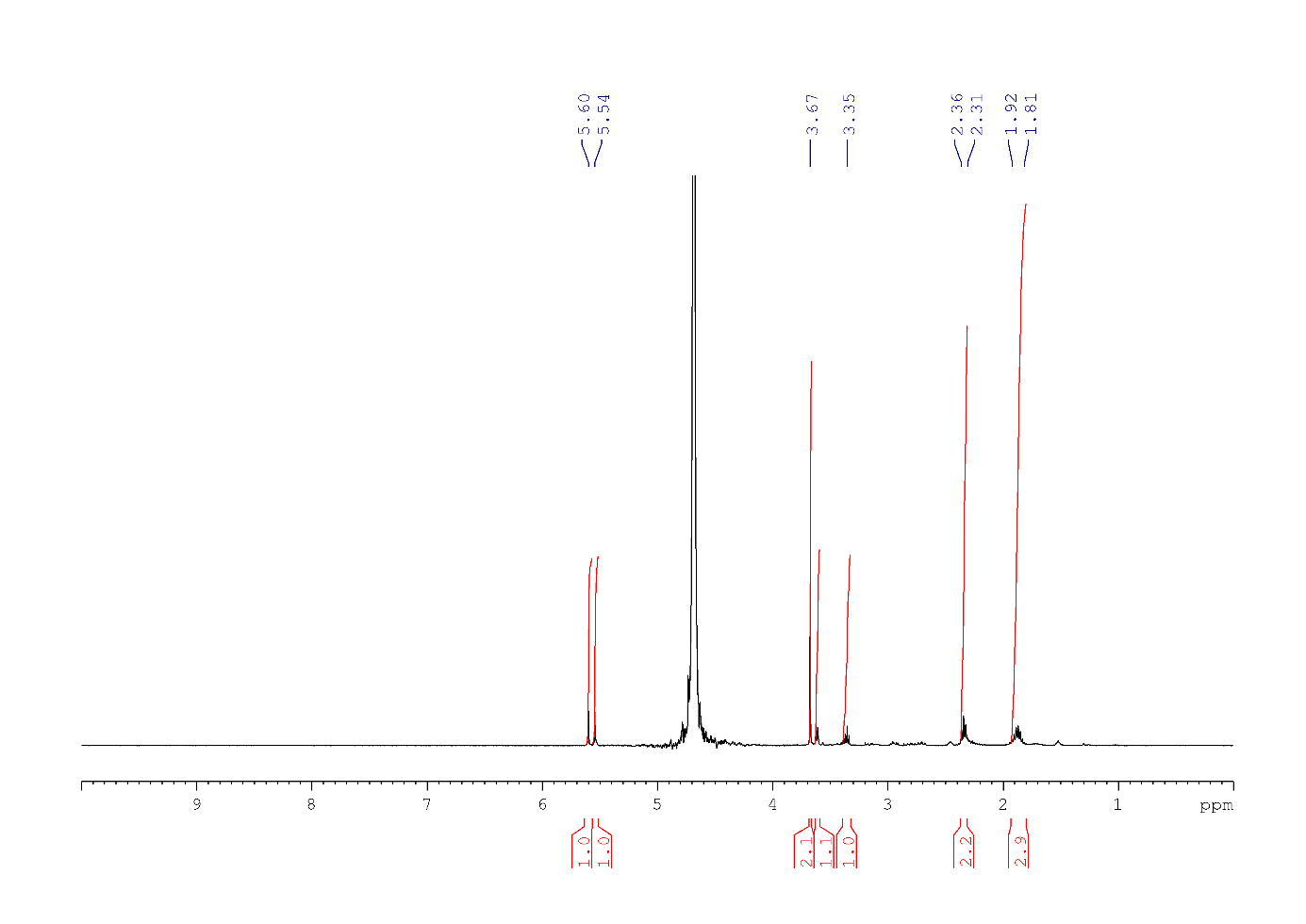
**


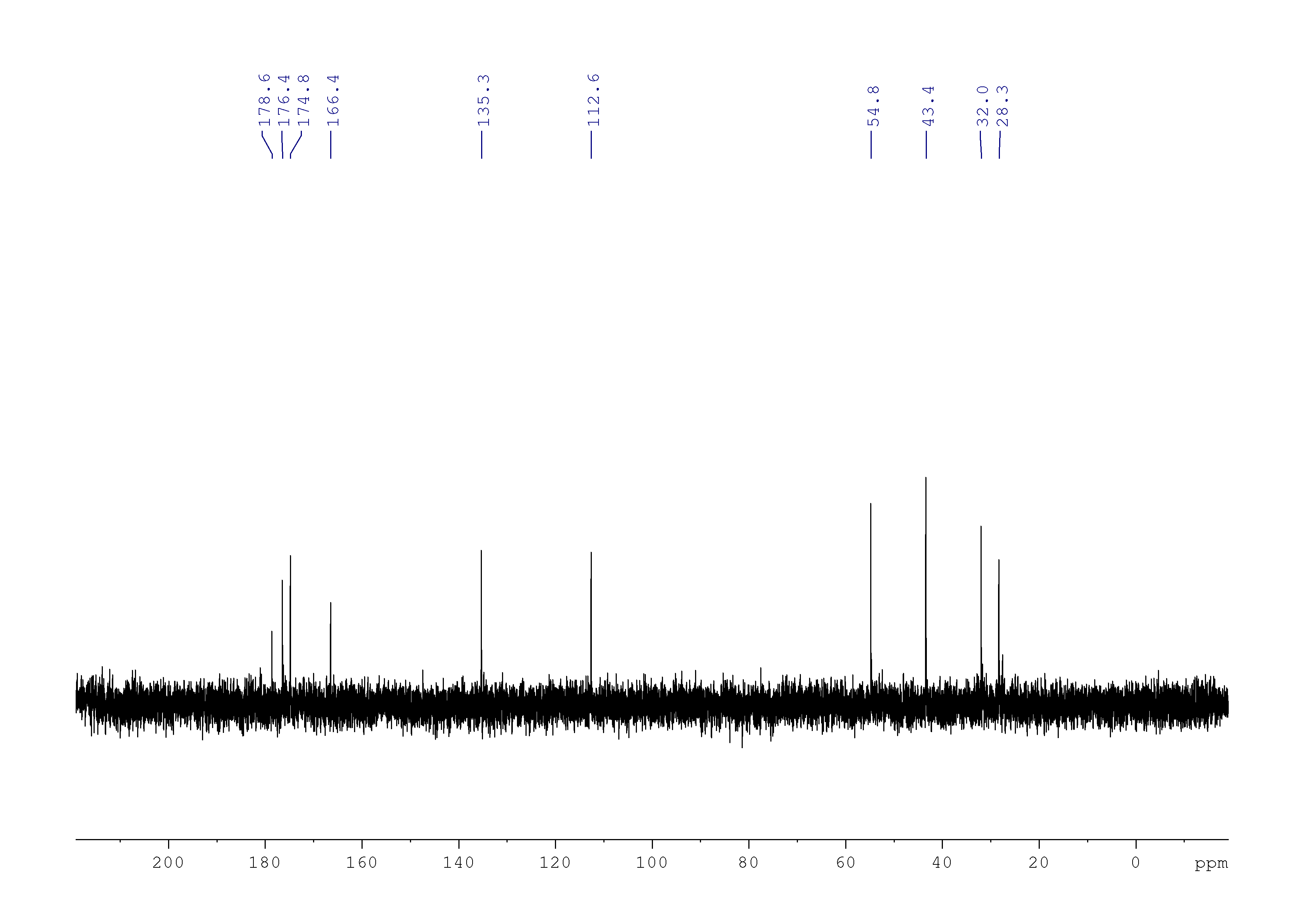


**16, CDCl_3_**

**
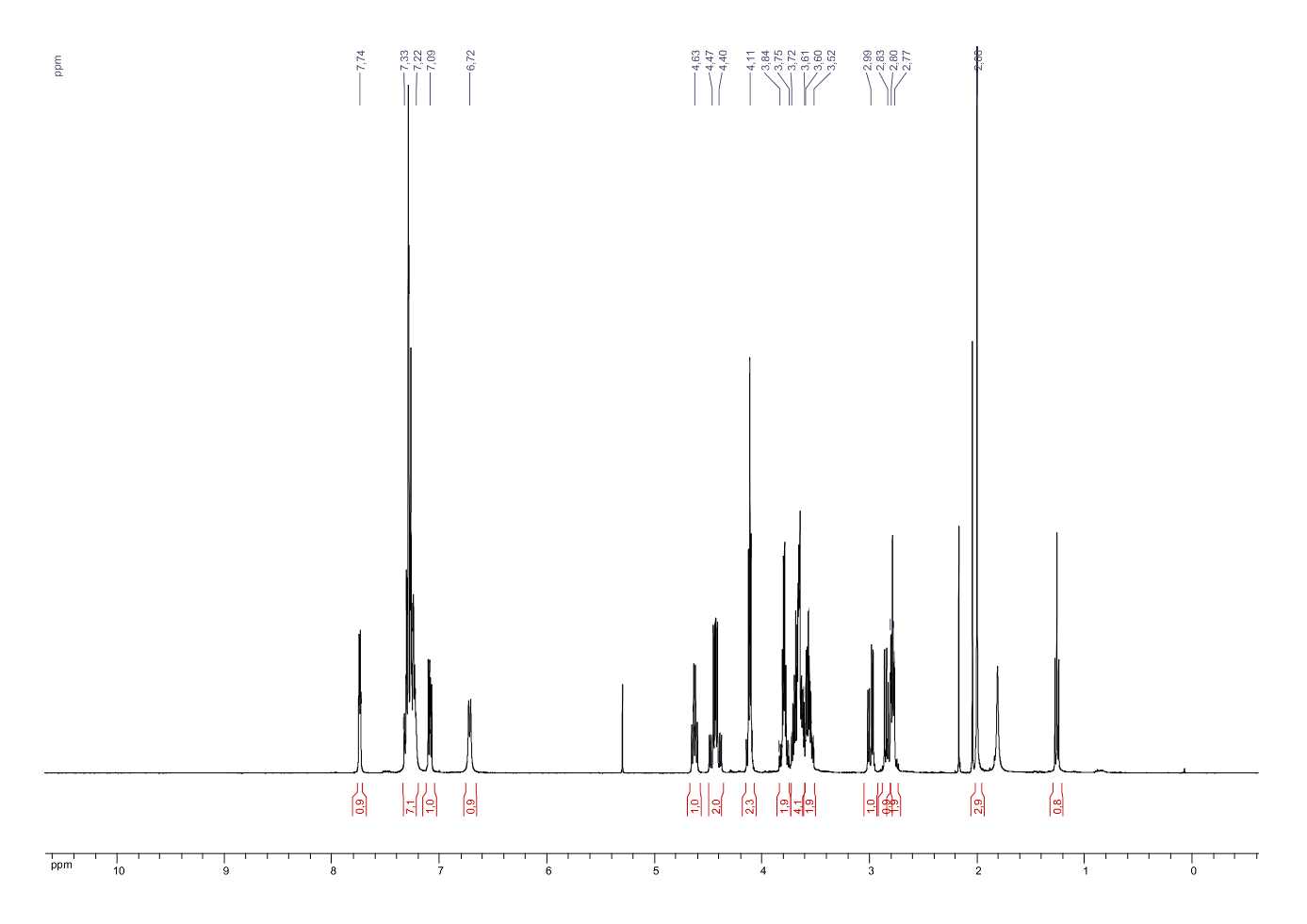
** **
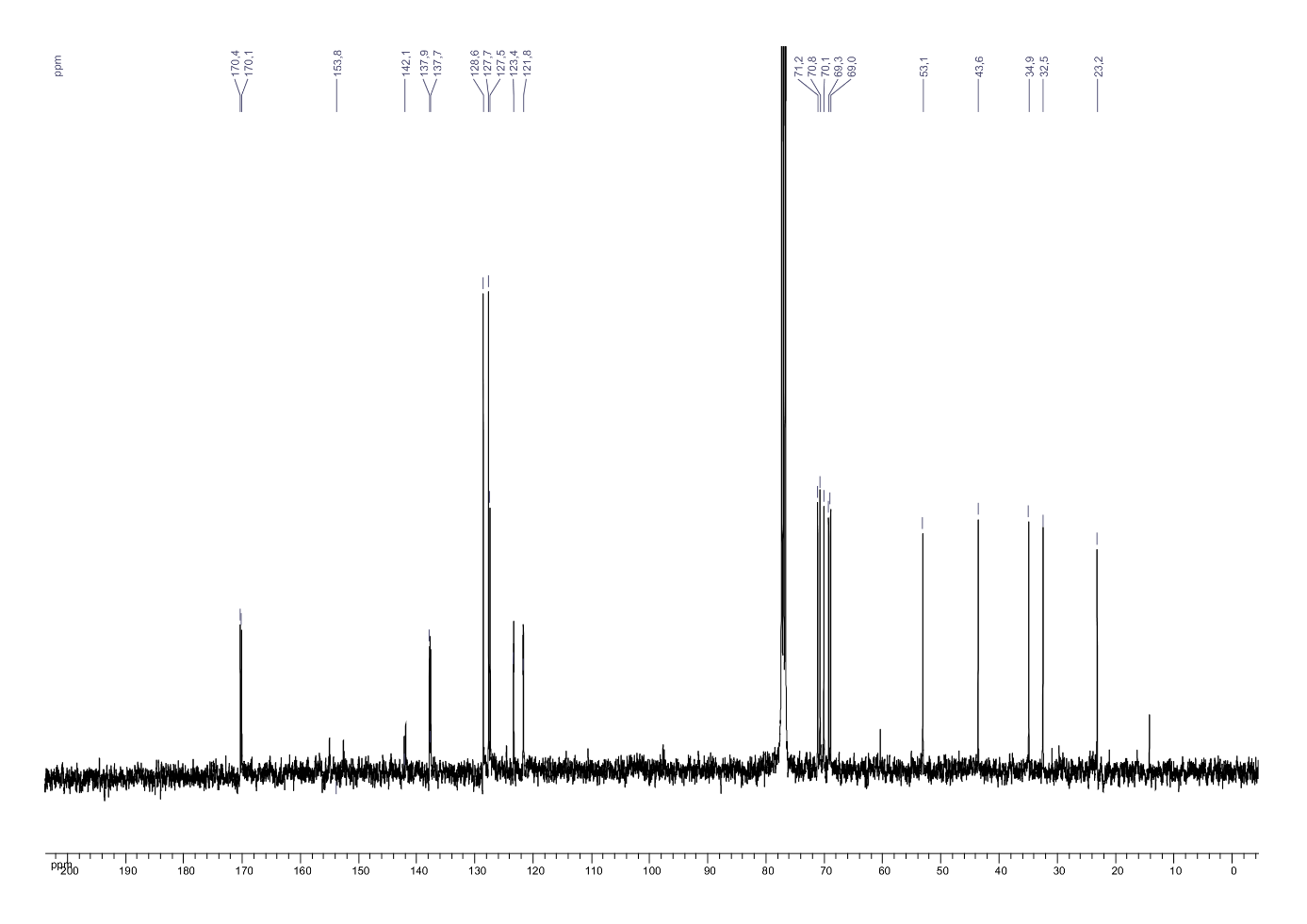
**

**17, D_2_O**

**
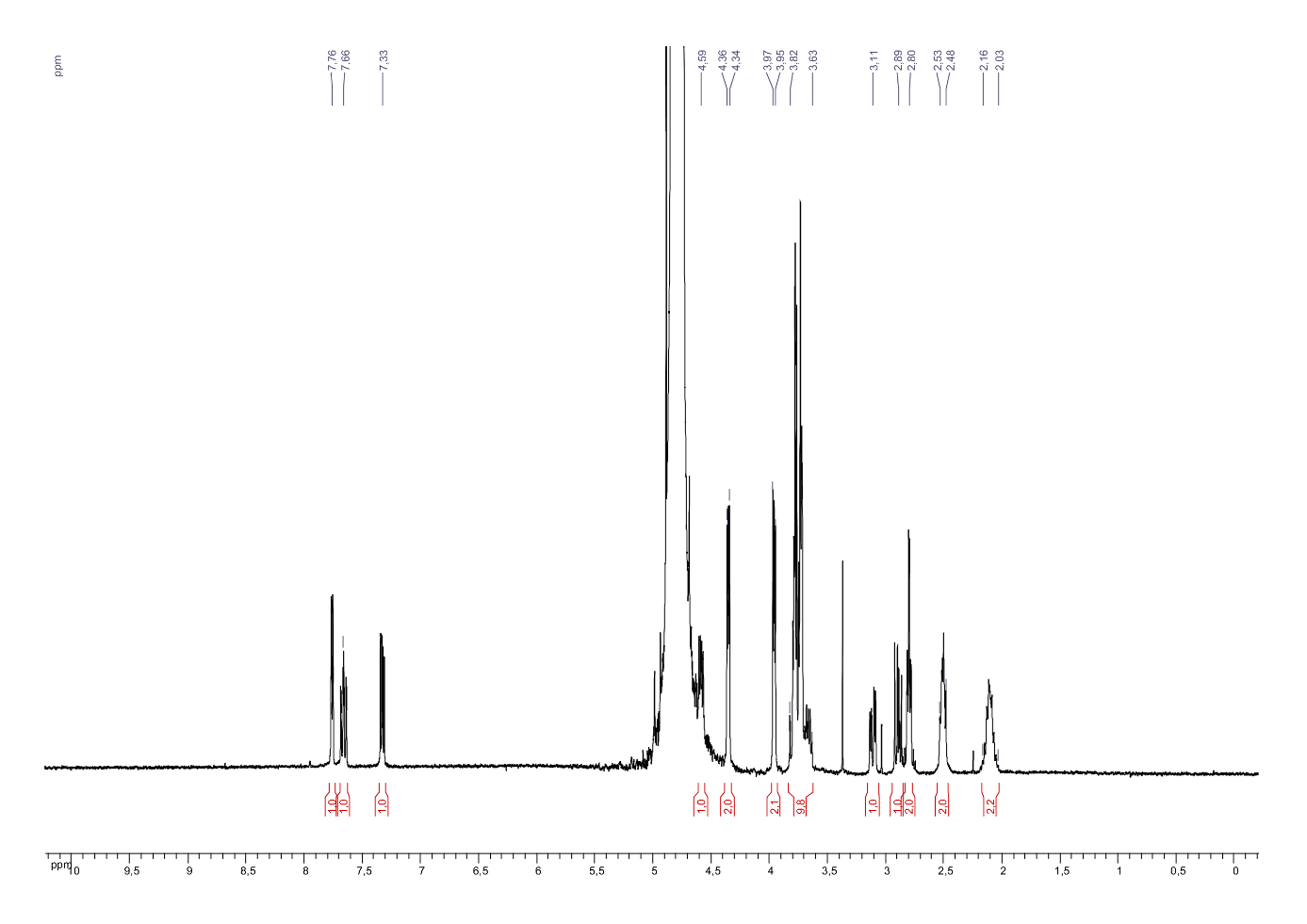
** **
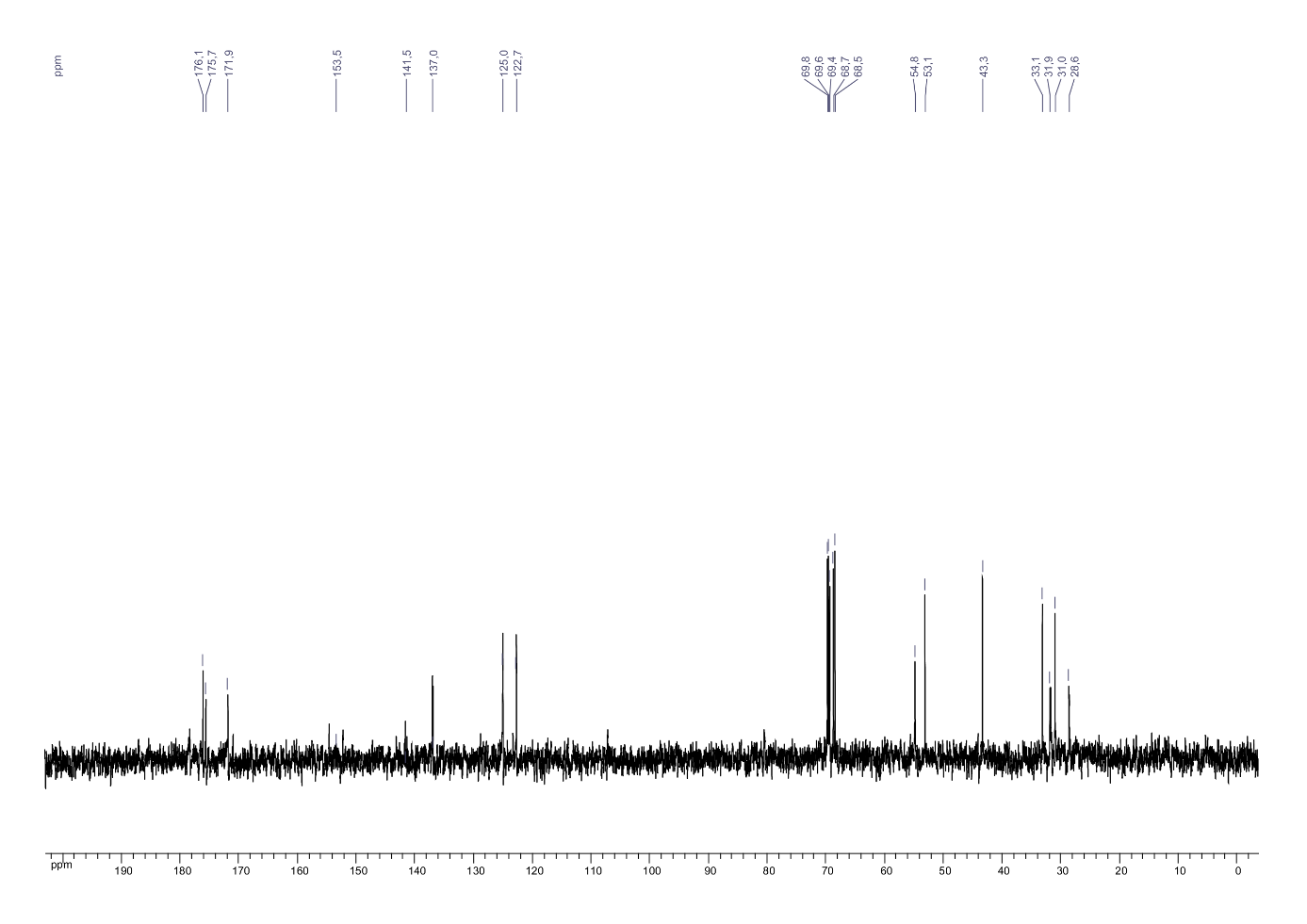
**

**15, D_2_O**

**
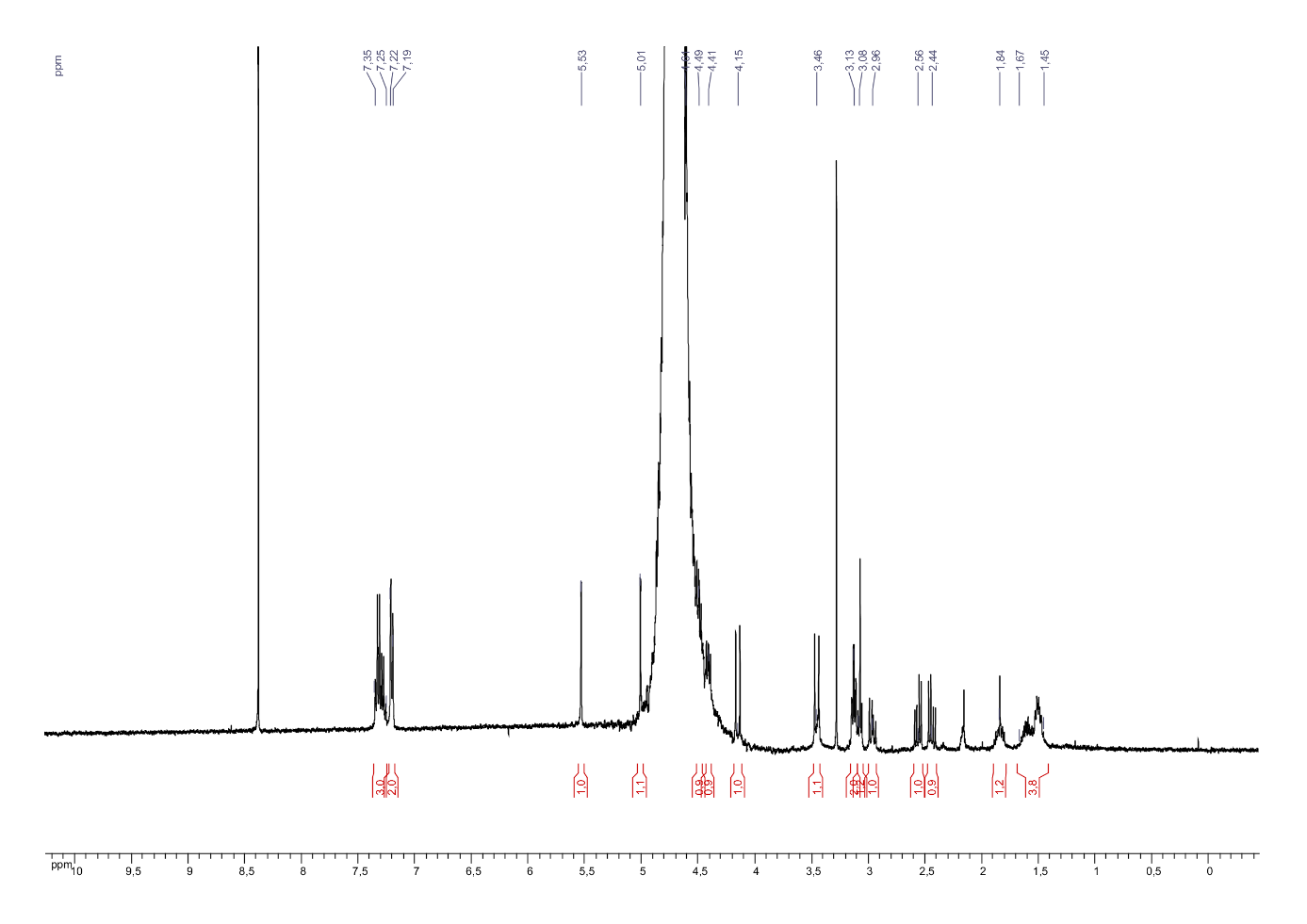
**

**18, D_2_O**

**
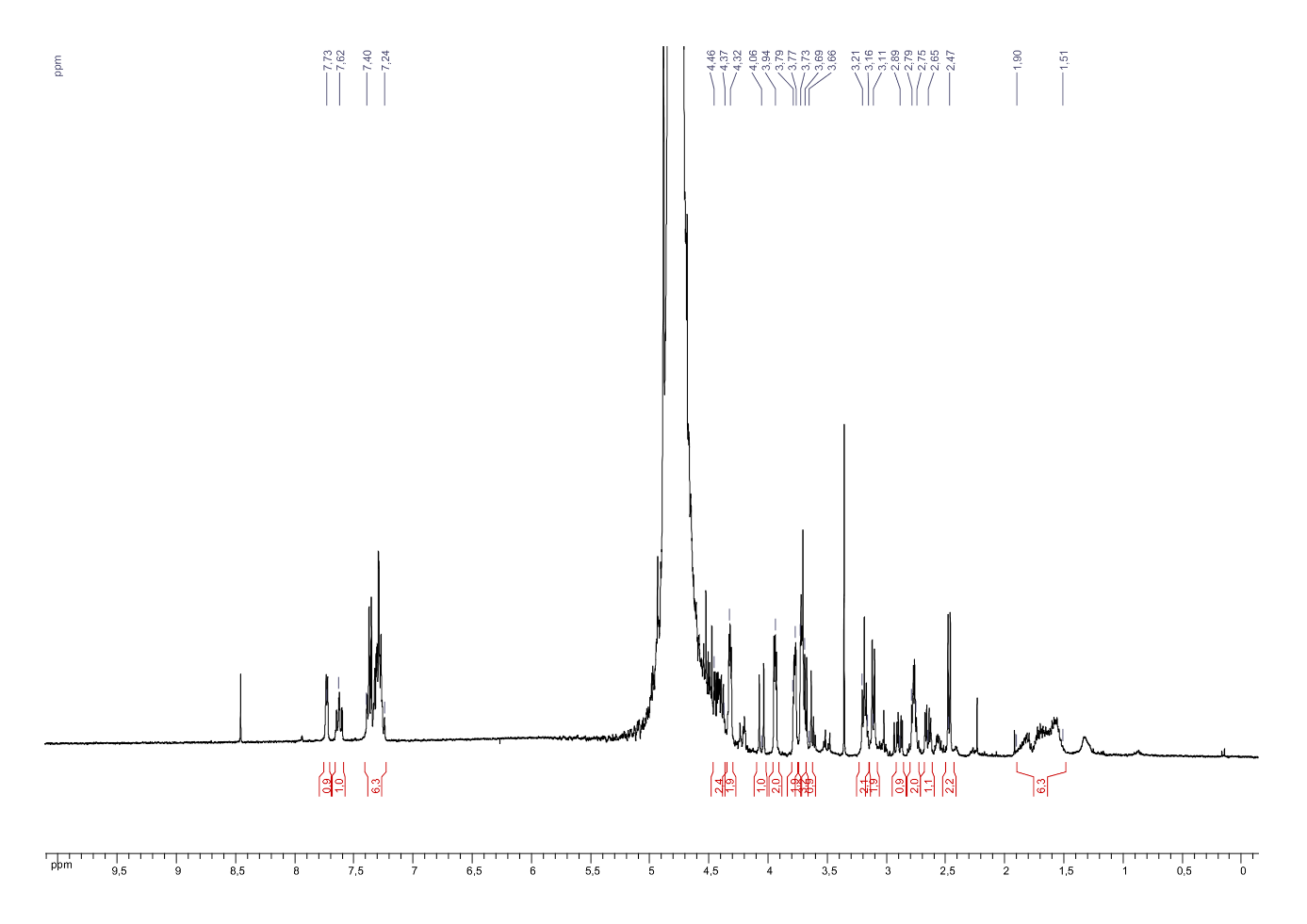
**

**20, D_2_O**

**
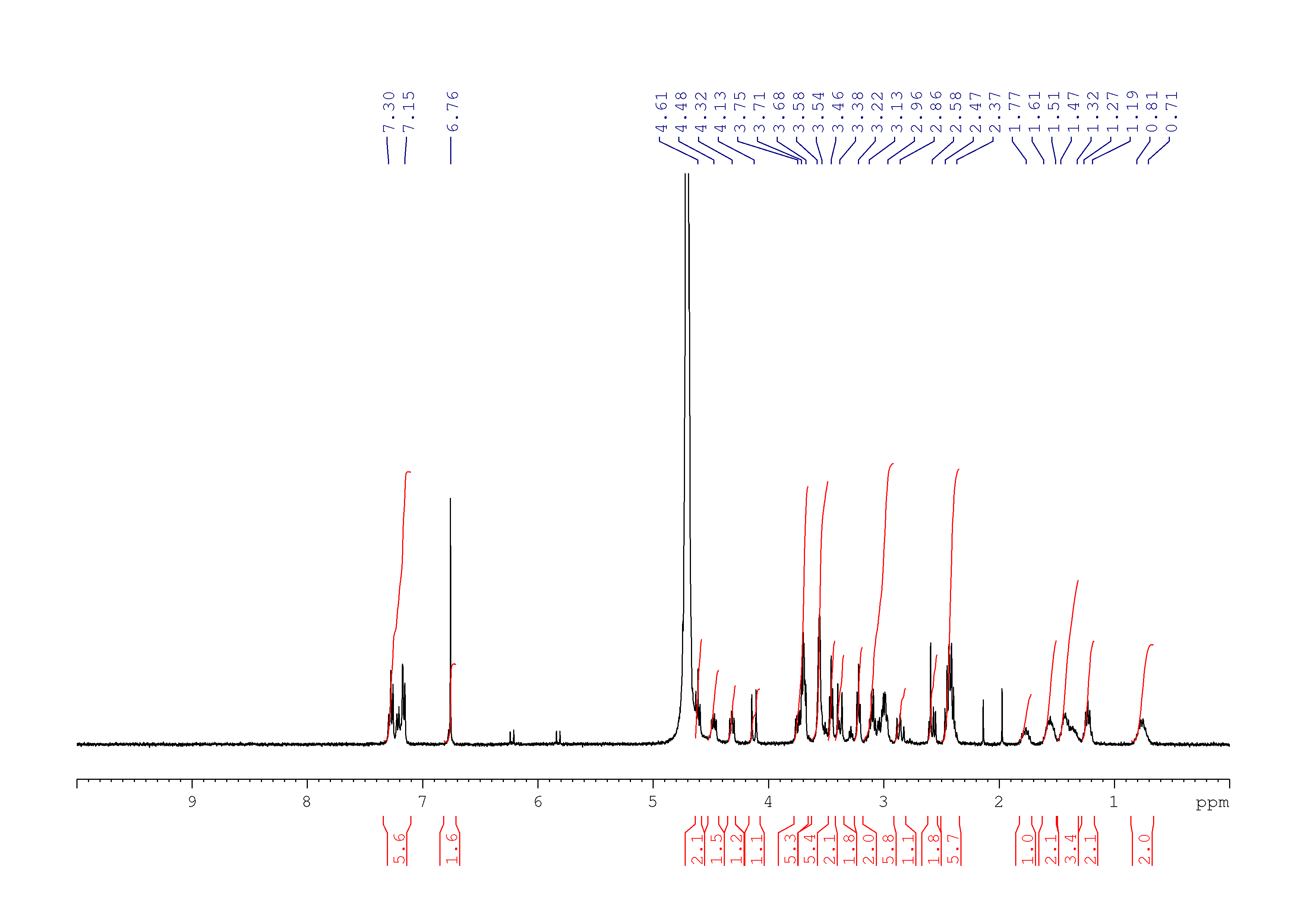
**

**21, D_2_O**

**
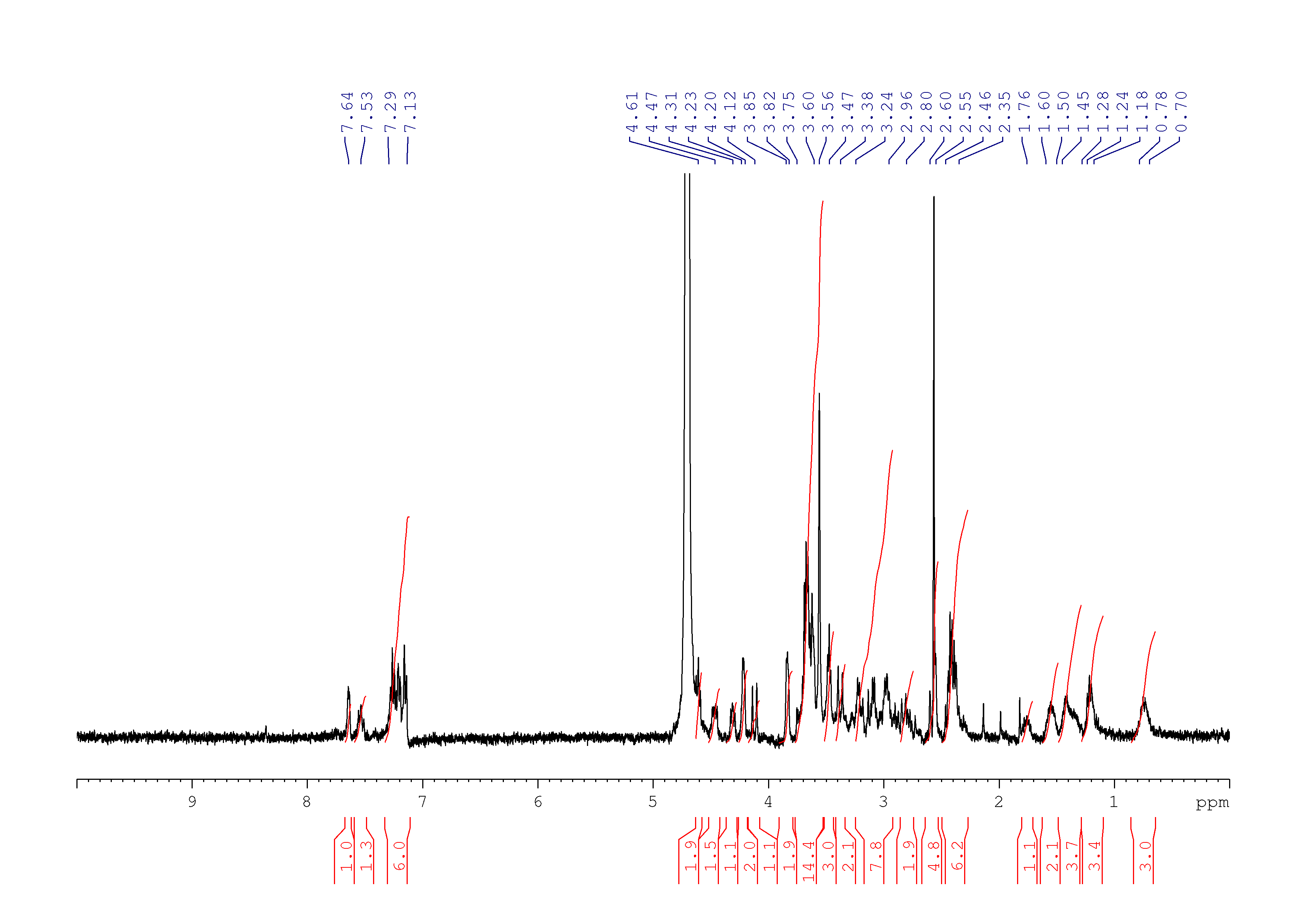
**

# III. Bibliography

Dedeoğlu B, Uğur İ, Değirmenci İ, Aviyente V, Barçın B, Çaylı G, et al. First RAFT polymerization of captodative 2-acetamidoacrylic acid (AAA) monomer: An experimental and theoretical study. Polymer. 2013;54(19):5122‑32.

Jukič M, Frlan R, Chan F, Kirby RW, Madge DJ, Tytgat J, et al. Synthesis and biological evaluation of piperazine derivatives as novel isoform selective voltage-gated sodium (Nav) 1.3 channel modulators. Med Chem Res. 2015;24(6):2366‑80.
